# SET-PP2A complex as a new therapeutic target in KMT2A (MLL) rearranged AML

**DOI:** 10.1038/s41388-023-02840-1

**Published:** 2023-10-27

**Authors:** Antonella Di Mambro, Yoana Arroyo-Berdugo, Tiziana Fioretti, Michael Randles, Luca Cozzuto, Vinothini Rajeeve, Armando Cevenini, Michael J. Austin, Gabriella Esposito, Julia Ponomarenko, Claire M. Lucas, Pedro Cutillas, John Gribben, Owen Williams, Yolanda Calle, Bela Patel, Maria Teresa Esposito

**Affiliations:** 1 School of Life and Health Sciences, University of Roehampton, London, UK; 2 CEINGE Biotecnologie Avanzate, Via Gaetano Salvatore, Napoli, Italy; 3 Chester Centre for Leukaemia Research, Chester Medical School, University of Chester, Chester, UK; 4 Centre Genomic Regulation (CRG), The Barcelona Institute of Science and Technology, Barcelona, Spain; 5 Barts Cancer Institute, Queen Mary University of London, London, UK; 6 Department of Molecular Medicine and Medical Biotechnologies, University of Naples Federico II, Via Pansini 5, 80131 Naples, Italy; 7 University Pompeu Fabra (UPF), Barcelona, Spain; 8 Great Ormond Street Institute of Child Health London, UCL, London, UK; 9 Present Address: School of Biosciences, University of Surrey, Guildford, UK

**Keywords:** Proteomics, Paediatric cancer, Transcriptomics, Acute myeloid leukaemia, Cell signalling

## Abstract

*KMT2A*-rearranged (KMT2A-R) is an aggressive and chemo-refractory acute leukemia which mostly affects children. Transcriptomics-based characterization and chemical interrogation identified kinases as key drivers of survival and drug resistance in *KMT2A*-R leukemia. In contrast, the contribution and regulation of phosphatases is unknown. In this study we uncover the essential role and underlying mechanisms of SET, the endogenous inhibitor of Ser/Thr phosphatase PP2A, in *KMT2A*-R-leukemia. Investigation of SET expression in acute myeloid leukemia (AML) samples demonstrated that SET is overexpressed, and elevated expression of SET is correlated with poor prognosis and with the expression of *MEIS* and *HOXA* genes in AML patients. Silencing SET specifically abolished the clonogenic ability of *KMT2A*-R leukemic cells and the transcription of *KMT2A* targets genes *HOXA9* and *HOXA10*. Subsequent mechanistic investigations showed that SET interacts with both KMT2A wild type and fusion proteins, and it is recruited to the *HOXA10* promoter. Pharmacological inhibition of SET by FTY720 disrupted SET-PP2A interaction leading to cell cycle arrest and increased sensitivity to chemotherapy in *KMT2A*-R-leukemic models. Phospho-proteomic analyses revealed that FTY720 reduced the activity of kinases regulated by PP2A, including ERK1, GSK3β, AURB and PLK1 and led to suppression of MYC, supporting the hypothesis of a feedback loop among PP2A, AURB, PLK1, MYC, and SET. Our findings illustrate that SET is a novel player in *KMT2A*-R leukemia and they provide evidence that SET antagonism could serve as a novel strategy to treat this aggressive leukemia.

## Introduction

Acute leukemias characterized by chromosomal translocations involving the *KMT2A* gene (previously known as *MLL*, *HRX, HTRX1, or ALL1*) are a highly aggressive group of leukemias with a poor prognosis [1, 2]. They account for over 80% of infant acute lymphoblastic leukemia (ALL), 5–10% of acute myeloid leukemia (AML), and over 70% of therapy related AML [2]. *KMT2A* encodes for the histone-lysine-N-methyltransferase 2A, which regulates gene transcription via methylation of histone H3 in the gene bodies [1, 3]. The N-terminal portion of KMT2A binds to DNA, whereas the C-terminal contains the catalytic domain for histone methylation, which is essential to relax the chromatin and to drive the expression of genes during embryogenesis and hematopoiesis [1]. *KMT2A* chromosomal translocations give rise to chimeric oncofusion proteins that retain only the N- terminal DNA binding domain of KMT2A fused with over hundred partner proteins [2]. These fusions impair the normal functioning of KMT2A and cause aberrant transcriptional activation by recruitment of an epigenetic multiprotein complex that includes the histone lysine 79 (H3K79) methyltransferase DOT1L, the bromodomain proteins, the acetyltransferase HBO1 and several other proteins that acquire opportunistic oncogenic functions [3–7]. The *HOX* genes, in particular *HOXA9* and *HOXA10*, are primary targets of *KMT2A*-fusion products and, together with the cofactor MEIS1, play an important role in the transcriptional reprogramming of hematopoietic stem cells (HSCs) and progenitor cells harboring the *KMT2A* translocations, leading to impairment of cellular differentiation [8–10] and resistance to DNA damage inhibitors [11]. In addition, expression of the oncogene *MYC* contributes to *KMT2A*-mediated leukemogenesis by driving cell proliferation and survival [12]. Despite the development of new therapeutic agents, such as inhibitors of KMT2A-fusion complex [3–5, 13–16] and inhibitors of kinases essential for the KMT2A-signaling pathways [17–19], none of these are FDA approved. Consequently, patients affected by *KMT2A-*R-leukemia are still treated with chemotherapy followed by bone marrow transplantation, with suboptimal outcomes. Therefore, the identification of novel targets and targetable pathways for *KMT2A*-R-leukemia is of primary importance for the development of new and effective therapeutic approaches.

SET is an oncoprotein also known as template activating factor-I-β, inhibitor 2 of Ser-Thr protein phosphatase 2A and putative histocompatibility leukocyte antigen class II-associated protein II. Originally identified as fusion gene in acute leukemia [20], it is overexpressed in several forms of solid tumors and hematological malignancies [21–26]. In *BCR::ABL*+ chronic myeloid leukemia (CML), SET-mediated PP2A inactivation is essential for the self-renewal of CML leukemic stem cells [27]. Silencing SET re-activates PP2A and switches the oncogenic driver kinase BCR::ABL off, highlighting its potency as a negative regulator of PP2A and a positive regulator of oncogenic cascades [21, 26, 28]. SET has also been recognized as a prognostic marker for poor overall survival in AML [22, 24], although the exact molecular mechanisms linking a SET oncoprotein pathway with aggressive AML outcomes remain still obscure. Interestingly, SET has been reported to interact with the N-terminal region of KMT2A, that is retained in KMT2A-fusion proteins [29]. However, the role of SET in the pathogenesis of *KMT2A*-R leukemia is unknown. Based on this and the fundamental role played by PP2A-modulated kinases on the survival and resistance of *KMT2A*-R-leukemic cells [17, 19, 30], we herein investigated the role of the PP2A endogenous inhibitor SET in *KMT2A*-R leukemia. Our overall results provide novel insights on the role of SET in *KMT2A*-R leukemia, via modulation of *HOXA* gene expression and MYC stability, and a proof of concept that inhibition of SET is a promising novel strategy to treat this aggressive form of acute leukemia.

## Results

### SET is over-expressed in *KMT2A*-R cell lines and primary samples

**Fig. 1:**
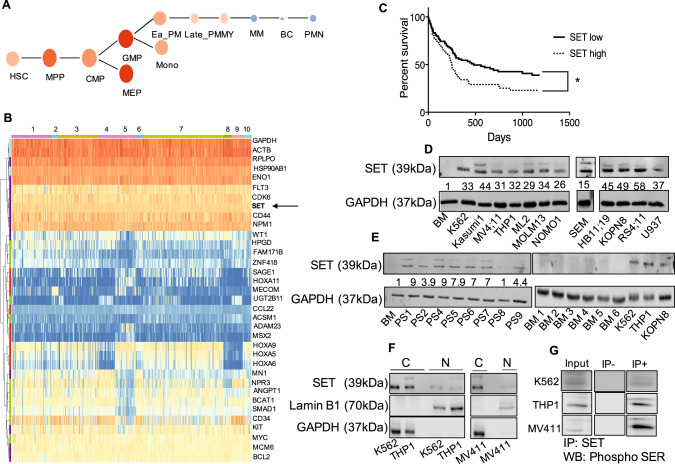
SET is an oncogene over-expressed in acute myeloid leukemia. **A** Hierarchical tree plot that shows the expression of *SET mRNA* in human HSCs, progenitors and differentiated blood cells. The level of expression is visualized by size and color of the nodes (data obtained from Bloodspot Gene expression profiles (GSE42519); *n* = 34; hematopoietic stem cells (HSC) n = 4, multipotential progenitors (MPP) *n* = 2, common myeloid progenitors (CMP) *n* = 3, granulocyte myeloid progenitors (GMP) *n* = 5, megakaryocyte-erythroid progenitor cells (MEP) *n* = 2, early promyelocyte (ea_PM) *n* = 3, late promyelocyte (late_PM) *n* = 3, myelocyte (MY) *n* = 2, metamyelocyte (MM) *n* = 3, band cell (BC) *n* = 4, polymorphonuclear cells *n* = 3. **B** Heatmap of *SET mRNA* along with other oncogenes, housekeeping genes and genes annotated as either down-regulated or up-regulated in *KMT2A*-R leukemia in a large AML- RNAseq dataset (*n* = 384) comprising 31 samples from patients carrying rearrangements of *KMT2A*. Meta-analyses of RNAseq data from Leucegene (GSE62190, GSE66917, GSE67039). 1: complex karyotype, 2: EVI-R; 3: intermediate; 4: inversion 16; 5: KMT2A-R; 6: monosomy 5; 7 normal karyotype; 8: *t*(15:17); 9: *t*(8;21); 10 trisomy-tetrasomy 8. **C** Kaplan–Meier analysis showing the survival in AML patients with low (*n* = 106) or high expression levels of SET (*n* = 57). Meta-analyses of micro-array data from the PrognoScan database (GSE12417). Log-Rank test; *p < 0.05. **D**, **E** Immunoblot of SET in *KMT2A*-R- AML cell lines (MV411, THP1, ML2, MLOM13, NOMO1), *KMT2A*-R-ALL cell lines (SEM, HB11;19, KOPN8, RS4;11), *KMT2A*-R-primary samples (PS) and six independent healthy bone marrow (BM) controls. The data also present the expression of SET in three *KMT2A*-wt cell lines K562 (*BCR::ABL* (t19;22) erythroleukemia cell line, Kasumi1 (*AML1:ETO, t8;21* AML cell line) and U937 (*CALM::AF10*, AML cell line). Densitometry analysis was conducted by Li-COR Image Studio software. GAPDH was used as a loading control. Values are expressed as ratio between SET and GAPDH, relative to the expression of SET in mononuclear cells isolated from bone marrow of healthy volunteers (BM). **F** Immunoblot of SET in cytoplasmic and nuclear factions of *KMT2A*-wt (K562) and *KMT2A*-R-AML cell lines (THP1 and MV411). Laminin B1 and GAPDH were used as nuclear and cytoplasmic markers, respectively. **G** Detection of ^Ser^SET in *KMT2A*-R cell lines. Immunoblotting against phosphorylated Ser was performed after SET immunoprecipitation.

We used public data repositories to characterize the role of SET across various hematopoietic contexts, to analyze its mRNA expression in human HSCs and progenitors (*n* = 34) [31], and in multiple independent human AML primary samples (*n* = 384) covering the main cytogenetic subsets [10]. SET was expressed at high levels in both HSC and myeloid progenitors (MP) compared with mature monocytes and myelocytes (Fig. 1A and Supplementary Fig. 1A), thereby indicating that it is a gene expressed during early hematopoietic development. In silico evaluation of SET mRNA expression across all AML was uniformly high and it did not show segregation with any distinct molecular group as previously suggested (Fig. 1B and Supplementary Fig. 1A–C) [22, 24]. A PrognoScan database-based Kaplan–Meier analysis of the overall survival of 163 AML patients by high (*n* = 57) and low (*n* = 106) SET levels [32], revealed that high SET expression positively correlated with poor overall survival in human AML (Fig. 1C and Supplementary Table 1), consistent with previous reports [24]. To further substantiate the role of SET in AML, we evaluated its protein levels by western blot in a panel of *KMT2A*-R-AML (THP1, MV411, ML2, MOLM13, NOMO-1) and *KMT2A*-R-ALL (SEM, Hb1119, KOPN8, RS411) cell lines and primary samples (PS), KMT2A wild-type (wt) cell lines (K562, *BCR::ABL*+ erythroleukemia, Kasumi1 *AML1::ETO* + AML, REH *TEL::AML* + ALL, U937 *CALM::AF10* + AML), mononuclear cells isolated from the bone marrow (BM) and peripheral blood (PB) of healthy adult volunteers. SET was significantly up-regulated in all leukemic cell lines and in all *KMT2A*-R primary samples, irrespective of tumor lineage, compared to BM controls (Fig. 1D, E). Given the poor outcomes of the *KMT2A*-R-leukemias and the relatively well characterized cellular context driving leukemogenesis, we further investigated the mechanistic role of SET in this group. As SET oncoprotein has several distinct roles depending on its subcellular localization [26, 33, 34], we analyzed its subcellular localization in two *KMT2A*-R-cell lines (THP1 and MV411) and one KMT2A-wt cell line (K562) by nuclear/cytoplasm fractionation followed by western blot and we showed that SET was relatively more abundant in the cytoplasm than in the nucleus of these cells (Fig. 1F). As phosphorylation of Ser9 and Ser25 of SET inhibits its nuclear import [26, 33, 34], we also investigated the phosphorylation status of SET. As no specific antibodies against phospho-SET are commercially available, SET was immunoprecipitated and protein samples were analyzed by western blot with anti-phospho-Ser antibodies. Consistent with the prevalent cytoplasmatic localization, our results showed that, in *KMT2A*-R-AML cell lines, SET is phosphorylated on serine residues (Fig. 1G and Supplementary 1D–F). Overall, these data indicate that SET is over-expressed, phosphorylated on Ser residues and abundantly localized in the cytosol in *KMT2A*-R and -wt leukemic cells.

### *SET* correlates with the expression of KMT2A targets *MEIS* and *HOXA* genes

**Fig. 2:**
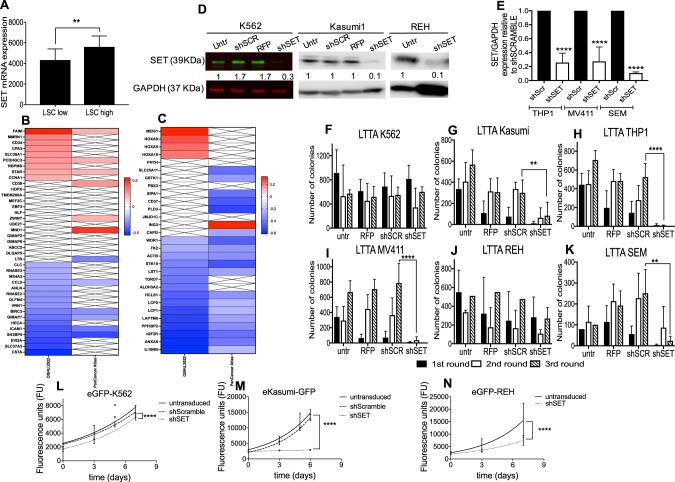
SET Knock down impairs the colony forming unity (CFU) ability of *KMT2A*-R-leukemic cells. **A** Expression of mouse *SET* mRNA (probe1421819_a_at) in 12 high LSC frequency *KMT2A-*R AMLs (*KMT2A::MLLT3* and *KMT2A:MLLT1*) and 22 low LSC frequency *KMT2A-*R-AMLs (*KMT2A::AFF1p*, *KMT2A::AF10* and *KMT2A::GAS7*). Meta-analyses of micro-array data from *GSE13690*. Unpaired *t* test; **p < 0.01. **B** Spearman correlation matrix for *SET* mRNA and human LSC genetic signature obtained from ref. [36]. **C** Spearman correlation matrix for *SET* mRNA and the self-renewal associated genetic signature identified in human KMT2A-R AML cells by [37]. The genes in red have a significant positive correlation with *SET* (Rs >0.2 and *p* < 0.05), while the genes in blue have a significant negative correlation with *SET* (Rs < −0.2 and *p* < 0.05). The genes marked with a cross do not correlate with *SET* expression. **D** Immunoblot for SET in K562, Kasumi1, and REH stably expressing shSCRAMBLE, RFP or shSET. Densitometry analysis was conducted by LI-COR Image Studio software. GAPDH was used as a loading control. Values are expressed as ratio between SET and GAPDH relative to untransduced cells. **E** qRT-PCR showing the expression of *SET* in eGFP-THP1, eGFP-MV411, and eGFP-SEM expressing either shScramble or shSET. Gene expression was normalized to *GAPDH* control and analyzed by Pfaffl equation. Values are expressed relative to shScramble controls. Data represent mean ± SD of three independent experiments. Unpaired *t* test **** *p* < 0.0001. **F–K** Effect of shSET on colony-forming unit ability. Data show mean ± SD of three independent experiments. 2-way Anova Tukey’s multiple comparative tests **p < 0.01; ****p < 0.0001. **K–N** Proliferation curve of K562 and Kasumi1 and REH, stably expressing eGFP and shScramble or shSET. GFP expression was used as quantitative reporter of cell proliferation. For each cell line, the same number of cells was plated at t0 and the GFP signal was measured by a fluorescent microplate reader at each time point. Data show mean ± SD of triplicate wells and are representative of three independent experiments. 2-Way Anova Tukey’s multiple comparison test *p < 0.05; **** *p* < 0.0001.

To understand the role of SET in leukemic stem cells (LSC), we analyzed the expression of *SET mRNA* in 12 high-LSC frequency mouse *KMT2A-*R AMLs and 22 low-LSC frequency *KMT2A-*R-AMLs, using a public dataset [35], as described in material and methods. Interestingly, we found that *SET* mRNA expression was significantly higher in high-LSC frequency *KMT2A*-R AML than in low-LSC frequency *KMT2A*-R AML (Fig. 2A), suggesting a potential role of SET in *KMT2A*-R leukemia self-renewal. We then used cBioPortal database to explore the potential correlation between the expression of *SET* mRNA and genes identified as LSC markers in human AML by Gentles et al. [36]. Out of the 52 genes identified by Gentles et al., 39 were found in the cBioPortal database for all the cohorts included in the study. Our in-silico analysis showed a significant positive correlation between *SET* expression and some LSC genes, such as *FAIM* and *STAR* (Fig. 2B). In addition, we checked whether there was a correlation between SET expression and the self-renewal associated genetic signature identified in *KMT2A*-R-AML by Krivtsov et al. [37]. In this case, out of the 34 described by Krivtsov et al., we found 29 genes in the cBioPortal database. *SET* expression significantly correlated with some primary targets of *KMT2A*-R-AML such as *MEIS*, *HOXA5*, *HOXA9* and *HOXA10* (Fig. 2C). These data indicate that SET expression is significantly higher in high-LSC frequency mouse KMT2A-R AML and that *SET* expression significantly correlated with the expression of some LSC marker genes and with KMT2A targets *MEIS* and *HOXA* genes.

### SET is essential for *KMT2A*-R leukemic cells’ clonogenic ability

To determine whether SET has a functional role in *KMT2A*-R- leukemia, we knocked down *SET* gene in human cell lines by using RNA interference. Analysis of protein by western blot (Fig. 2D) and of gene expression by RT-qPCR (Fig. 2E) confirmed the knock down (KD) in all the cell lines investigated. We then assessed the effect of SET KD on the clonogenic ability of the cells. *SET* KD completely abolished the clonogenic ability of *KMT2A*-R leukemic cell lines (THP1, MV411, and SEM, Fig. 2H, I and K and Supplementary Fig. 2), whereas it had little or no effect on the colony formation of three independent *KMT2A*-wt leukemic cell lines (K562, Kasumi1, and REH Fig. 2F, G, and J). Then, we determined the impact of SET KD on cell proliferation of KMT2A-wt leukemic cells by monitoring GFP fluorescence and by MTS assay, as described in [38] and in Supplementary material and methods. The correlation between the GFP fluorescence and the OD values determined for the MTS assay are reported in supplementary Fig. 3. The correlation between two assays were above 0.8, indicating that the GFP monitoring of the newly generated GFP-expressing lines can be used as a readout of cell proliferation in these experimental conditions. SET KD attenuated the proliferation of K562 (Fig. 2L, Supplementary Fig. 3 and Supplementary Table 2), in line with a previous report [21]. SET KD had a stronger impact on *KMT2A*-wt AML cell line Kasumi1 (Fig. 2M, Supplementary Fig. 3 and Supplementary Table 2) and on *KMT2A*-wt ALL cell line REH (Fig. 2N, Supplementary Fig. 3 and Supplementary Table 2). Interestingly, whereas the GFP monitoring indicates that eGFP-Kasumi1-shSET proliferate significantly more slowly than eGFP-Kasumi1-shScramble, the MTS assay indicates that eGFP-Kasumi-shSET are still metabolically active. The discrepancy between these results might be due to the emergence of growth arrested cells that are still highly metabolically active [39]. Overall, these data indicate that SET-KD specifically abolishes the clonogenic ability in *KMT2A*-R-leukemic cells and it attenuates the proliferation of *KMT2A-*wt leukemic cells; whereas in K562 the effect on proliferation is mild, in Kasumi1 and REH SET KD induces a significant arrest in proliferation.

### The SET inhibitor FTY720 induces cell cycle arrest and drives cell death in *KMT2A*-R leukemic cells

**Fig. 3:**
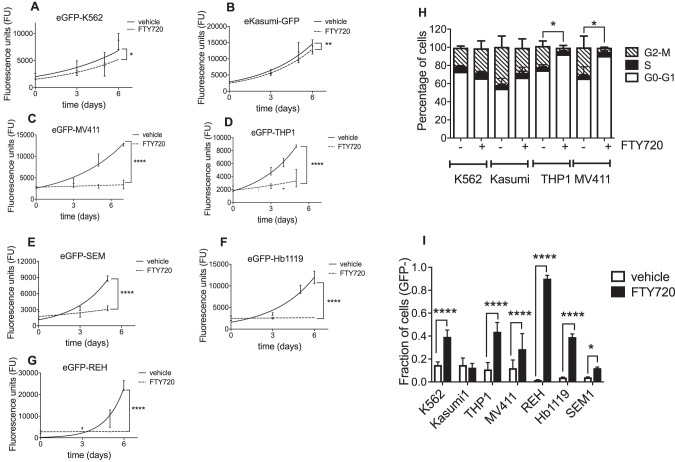
FTY720 induces cell cycle arrest and drives apoptosis in *KMT2A*-R leukemic cell lines. **A–G** Proliferation curve of K562, Kasumi, THP1, MV411, SEM, Hb1119, and REH stably expressing eGFP, upon treatment with FTY720 for 6 days. GFP expression was used as quantitative reporter of cell proliferation. For each cell line, the same number of cells was plated at t0 and the GFP signal was measured with a fluorescent microplate reader every 2 days. Data show mean ± SD of triplicate wells and are representative of three independent experiments. 2-Way Anova Sydak’s multiple comparison test *p < 0.05; ** *p* < 0.01; **** *p* < 0.0001. **H** Cell cycle analysis performed on K562, Kasumi1, MV4,11 and THP1, upon 5 µM FTY720 treatment for 48 h. Data show mean ± SD of triplicate wells and are representative of two independent experiments. Two-tailed unpaired *t* test *p < 0.05. **I** Fraction of cells undergoing cell death (GFP-) upon 5 µM FTY720 treatment for 48 h. Data show mean ± SD of triplicate wells and are representative of three independent experiments. Two-tailed unpaired *t* test *p < 0.05; ****p < 0.0001.

We next tested pharmacological modulation of SET by FTY720 (Fingolimod) [40], a FDA-approved immunosuppressive drug that has gained further attention as anti-cancer and PP2A activating drug, due to its ability to disrupt the binding between SET and PP2A [28, 41–43]. We first carried out a dose-response titration assay and determined that the half maximal inhibitory concentration of FTY720 in vitro ranged between 1 and 5 μM (Supplementary Fig. 4A), which were reported as non-toxic to healthy bone marrow mononuclear cells [27, 28]. We then assessed the effect of FTY720 on proliferation, cell cycle and cell death of *KMT2A*-wt (K562, Kasumi1 and REH) and *KMT2A*-R-leukemic cells (THP1, MV411 Hb119, and SEM) (Fig. 3A–G and Supplementary Fig. 3 and Table 2). We observed that 5 µM FTY720 had a variable but significant effect on the proliferation of all the analyzed cell lines, ranging from modest for the *KMT2A*-wt-cell lines K562 and Kasumi1 to severe for eGFP-REH (Fig. 3G and Supplementary Fig. 3 and Table 2), as reported [28, 44, 45]. In addition, FTY720 severely halted the proliferation of all the *KMT2A*-R-cell lines. Cell cycle analyses revealed that treatment with FTY720 for 48 h induced a significant increase of cells in G1 and a reduction in S and G2-M phase, in *KMT2A*-R-cells (Fig. 3H and Supplementary Fig. 4B), indicating cell cycle failure. Moreover, we investigated the fraction of leukemic cells undergoing cell death upon FTY720 treatment by FACS, by gating the GFP negative (GFP-) cells, as described previously [38] and in the Supplementary material and methods. FTY720 induced a statistically significant increase in cell death in two *KMT2A*-wt-cell lines K562 and REH whereas this was not significant for the *KMT2A-*wt cell line eGFP-Kasumi1. In contrast, FTY720 induced a consistent and statistically significant increase in cell death in *KMT2A*-R-cells (Fig. 3I and Supplementary Fig. 4C). These results indicate that the SET inhibitor FTY720 induces heterogenous effects in leukemic cells; in K562 FTY720 has a modest effect on proliferation and cell death; in Kasumi1 FTY720 has a modest effect on proliferation and it does not induce cell death; in REH, FTY720 significantly impairs the proliferation and it induces cell death; in *KMT2A*-R-leukemic cells, FTY720 significantly impairs the proliferation, causes cell cycle arrest in G1 and increases the rate of cell death.

### The effect of FTY720 is dependent on PP2A activation

**Fig. 4:**
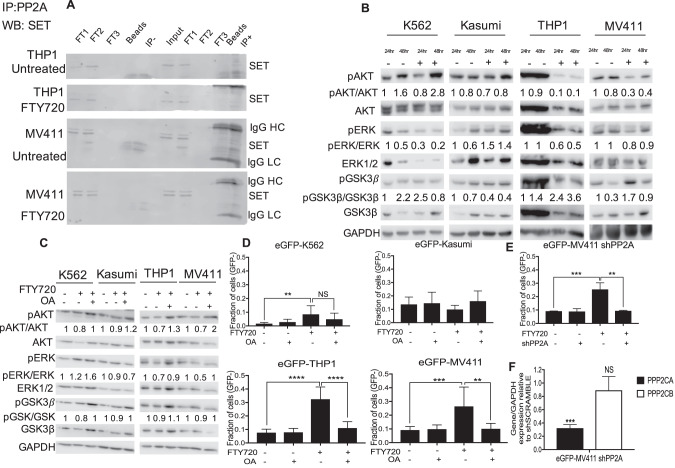
The Effect of FTY720 on *KMT2A*-R cells is dependent on PP2A activation. **A** Protein complex -immunoprecipitation showing SET—PP2A interaction in *KMT2A*-R- AML cell lines upon FTY720 treatment for 24 h. Immunoblotting against SET (39 kDa) was performed after Immunoprecipitation of PP2A (FT: Flow through control; IgG HC: IgG high chains; IgG LC: IgG low chains). **B** Immunoblot for phospho-AKT1/2 (Ser473) (60 kDa), AKT (pan)(60 kDa), phosphoGSK3β (Ser9) (42 kDa), GSK3β (42 kDa), phosphoERK1/2 (Thr402/Tyr404) (42–44 kDa), ERK1/2 (42–44 kDa) and GAPDH (37 kDa) in K562, Kasumi1, THP1 and MV411 upon 5 µM FTY720 treatment for 24 and 48 h. Densitometry analysis was conducted by Li-cor Image Studio software. GAPDH was used as a loading control. Values are expressed relative to the vehicle at 24 h. **C** Immunoblot for phospho-AKT1/2 (Ser473) (60 kDa), AKT (pan) (60 kDa), phosphoGSK3β (Ser9) (42 kDa), GSK3β (42 kDa), phosphoERK1/2 (Thr402/Tyr404) (42–44 kDa), ERK1/2 (42–44 kDa) and GAPDH (37 kDa) in K562, Kasumi1,THP1 and MV411 upon 5 µM FTY720 treatment for 24 h. Cells were pre-treated with 2.5 nM Okadaic Acid for 4 h. Densitometry analysis was conducted by LI-COR Image Studio software. GAPDH was used as a loading control. Values are expressed relative to the vehicle. **D** Analysis of cell death upon FTY720 treatment. Cells were pre-treated with 2.5 nM Okadaic Acid for 4 h and then treated with 5 µM FTY720 for 72 h. GFP signal was used as quantitative reporter of alive, non-apoptotic cells and measured by fluorescent- activated cell sorting (FACS). Data show mean ± SD of triplicate wells and are representative of three independent experiments. 2-Way Anova Dunet’s multiple comparison test **p < 0.01; ***p < 0.001; ****p < 0.0001. **E** Analysis of cell death of eGFP-MV411 transfected with either shSCRAMBLE or shPP2A upon FTY720 treatment for 72 h. GFP signal was used as quantitative reporter of alive cells and measured by flow cytometry. Data show mean ± SD of triplicate wells and are representative of three independent experiments. 2-Way Anova Tukey’s multiple comparison test **p < 0.01; ***p < 0.001. **F** qRT-PCR showing the expression of *PPP2CA and PPP2CB* in eGFP-MV411 shPP2A. Gene expression was normalized by *GAPDH* control and analyzed by Pfaffl equation. Values are expressed relative to shSCRAMBLE controls. Data represent mean ± SD of three replicas. Two tailed paired *t* test *** *p* < 0.001.

FTY720 is a SET inhibitor able to rescue the activity of PP2A towards its target pathways [40, 46]. By immunoprecipitation, we showed that FTY720 treatment for 24 hours disrupted the binding between SET and PP2A in *KMT2A*-R cells, confirming the molecular mechanism reported in other leukemic models (Fig. 4A) [28, 41–43]. To investigate whether the observed effects of FTY720 on *KMT2A*-R cells were due to the activation of PP2A, we analyzed the phosphorylation of some of PP2A targeted pathways [47] by western blot. In K562 cells, we observed reduction in phospho-ERK1/2 (Thr202/Tyr204), which was instead unchanged in Kasumi1; in these two cell lines, phospho- GSK3β (Ser9) expression decreased, upon FTY720 treatment. Notably, in *KMT2A*-R-leukemic cells, we observed a dramatic and stable decrement in the expression of the PP2A target phospho- ERK1/2 (Fig. 4B). In addition, we observed a sustained reduction in the abundance of phospho-AKT1(Ser473), a phosphosite identified as marker of active AKT1 (Fig. 4B). To confirm that the effect of FTY720 was specifically dependent on PP2A activation, we performed the same experiments by pre-treating cells with the phosphatase inhibitor okadaic acid (OA). We first tested OA in K562 cells and confirmed that the range of concentrations used did not impact the cell proliferation (Supplementary Fig. 5A). Treatment with 5 nM OA for 2 hours induced a significant increase in pAKT Ser473, a phosphosite that is not reported as regulated by PP2A (PhosphositePlus), indicating that this concentration might alter the activity of other phosphatases. At lower concentrations, equal to 2.5 nM, OA caused a significant increase in three PP2A targets, phospho-GSK3β Ser9, phospho-ERK1 (Thr202/Tyr204) and phospho-AKT Thr308 with maximal expression after 2–4 hours of treatment (Supplementary Fig. 5B, C), indicating that, at this concentration, OA preferentially inactivates PP2A. We therefore used this concentration of OA for our combination experiments with FTY720. Western blot analysis of PP2A targets in the cells pre-treated with OA for 4 h and then treated with FTY720 for 20 hours, revealed that pre-treatment with OA restored phospho-AKT and phospho-ERK1/2 levels in KMT2A-R- leukemic cells (Fig. 4C). Whereas pre-treatment with OA did not have any effect on the percentage of dead cells in eGFP-K562 and eGFP-Kasumi1, it significantly decreased the percentage of cell death in *KMT2A*-R-cells (Fig. 4D and Supplementary Fig. 6), suggesting that OA rescues the effect of FTY720 in *KMT2A*-R-leukemic cells. A similar effect was obtained by knocking down *PPP2CA*, the gene encoding for the catalytic α subunit of PP2A (Fig. 4E, F). These data indicate that the effects of FTY720 on *KMT2A*-R cells are dependent on PP2A activation.

### The phospho-proteome reveals FTY720-mediated effects on cell division, apoptosis and gene transcription

**Fig. 5:**
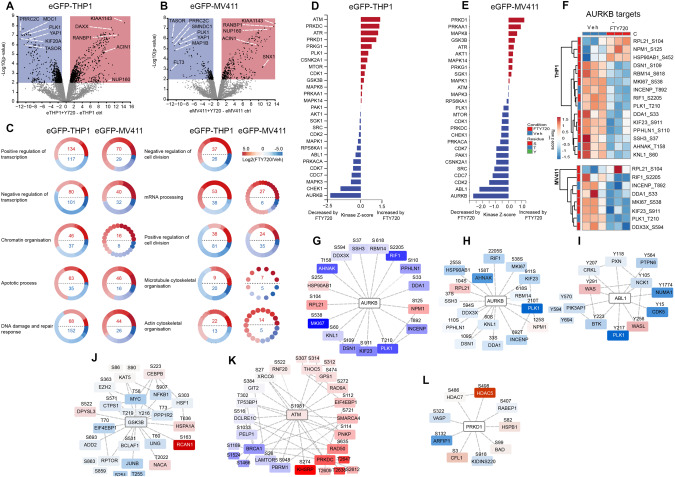
Phospho-proteomic profile analysis of FTY720 treated *KMT2A*-R leukemic cells. Characterization of the impact of FTY270 on kinase signaling networks in eGFP-THP1 and eGFP-MV411 cells. **A**, **B** Volcano plot showing the most highly enriched phosphosites from the Phospho-proteomic analysis of cells treated with 5 μM FTY720 for 48 h relative to vehicle. Color coded quadrants represent phopshopeptides that possess a fold change >2 and *p* value < 0.01. **C** Over-representation analyses of Gene Ontology Biological Processes (GO_BP) enriched in cells treated with 5 μM FTY720 for 48 h relative to vehicle. Representative labeled terms were determined using affinity propagation, nodes represent individual phophoproteins allocated to the GO_BP and color coded to represent Log2(FTY720/Vehicle). Attribute circle layout was used based on Log2(FTY720/Vehicle) and total numbers of enriched phosphoproteins are shown within the center circle for each term. **D**, **E** Kinase-Substrate Enrichment Analysis (KSEA) of cells treated with 5 μM FTY720 for 48 h relative to vehicle. KSEA was performed using KSEA App. For a given upstream kinase, the m threshold was set to 5, the NetworKIN PhosphoSitePlus threshold was set to 5 and the *p* value cut off was <0.1. **F** Hierarchical clustering of phosphosites allocated to specific kinases by KSEA analysis. To create groups of phosphorylation sites that share similar patterns of abundance changes targets of specific kinases were grouped together by KSEA analysis. Log2 fold-change of >1 and *p* value < 0.1 filters were applied to exclude phosphosites that were unchanged in abundance and phosphosite abundances were z-transformed by row. Euclidean distance average linkage clustering was then applied to these datapoints and clustered heatmaps shown. **G**–**L** Interaction network analysis. Phosphosites allocated to kinases with enriched or inhibited activity due to FTY270 were mapped via network interaction analysis. Nodes represent proteins with their phosphosites mapped on. Color code represents Log2(FTY720/Vehicle) and edges represent known protein-protein interactions. **G**, **K** show data for eGFP-THP1 cells and **H**, **I**, **J** and **L** show data for eGFP-MV411 cells.

To reveal the global impact of FTY720 on leukemic cells, we performed a phospho-proteomic analysis on two *KMT2A*-R cell lines (eGFP-THP1 and eGFP-MV411) treated with FTY720, using in gel digestion and liquid chromatography–tandem mass spectrometry (LC–MS/MS) [48]. LC–MS/MS analysis showed a differential pattern of phospho-protein abundance in eGFP-THP1 and eGFP-MV411 cells, with 2276 phosphosites up-regulated and 1862 phosphosites down-regulated (>2 fold and *p* < 0.01) in eGFP-THP1, and with 1428 phosphosites up-regulated and 743 phosphosites down-regulated (>2 fold and <0.01) in eGFP-MV411 (Fig. 5A and B and Supplementary Fig. 7), in comparison to vehicle-treated cells. The complete list of identified proteins and phospho-peptides are provided in Supplementary Table 3. To gain deeper biological insights, we categorized the phospho-proteins using Gene Ontology (GO) (Fig. 5C), revealing that treatment with FTY720 led to a robust decrease in cell division and an increase in apoptosis-related kinase signaling in eGFP-THP1 (Fig. 5C and Supplementary Fig. 7E). In contrast, the data indicate a strong decrease in cell division-related kinase signaling, but a subtler increase in apoptosis eGFP-MV411 (Fig. 5C and Supplementary Fig. 7F). In addition, FTY720-modulated phosphosites implicated in transcription regulation, chromatin organization, DNA damage repair, mRNA processing, microtubule and actin cytoskeletal organization (Fig. 5C). We then used Kinase-Substrate Enrichment Analysis (KSEA) for the characterization of kinase activity from the phospho-proteomic dataset. The mitosis-regulating kinase Aurora kinase B (AURB), a critical regulator of MYC protein stability and transcriptional activity [49], was the kinase most significantly impacted by FTY720 in both cell lines (Fig. 5D, E). Indeed, FTY720 decreased the phosphorylation of several AURB targets, including PLK1 (Thr210) (Fig. 5F, G, H). As expected, FTY720 inhibited MAPK3 (ERK1) and phosphorylation of ERK1 on Thr202 and Tyr204 was significantly reduced in both cell lines (Supplementary Fig. 7G); furthermore, FTY720 significantly inhibited Abl1 and CDK2 in eGFP-MV411 (Fig. 5I and Supplementary Fig. 7I). Although the overall effect of FTY720 resulted in a significant increase in GSK3β activity, the analysis of the phosphosites regulated by this kinase indicated that this effect was mostly due to the increased phosphorylation of a single target RCAN1 (Ser163), whereas GSK3β-dependent phosphorylation of MYC on Thr58 was significantly inhibited by FTY720 (Fig. 5J and Supplementary Fig. 7J). FTY720 significantly increased the activity of DNA damage kinase ATM, with an overall activation of targets implicated in DNA repair by non-homologous end joining (NHEJ), such as PRKDC (DNA-PK), and inhibition of sensors involved in transcription and DNA repair by homologous recombination (HR), such as BRCA1 (Fig. 5K, L and Supplementary Fig. 7K). Taken together, these data indicate that FTY720 reduced the activity of phospho-signaling associated to cell division and MYC stability and increased apoptosis-related and DNA damage kinase signaling in *KMT2A*-R cells.

### FTY720 affects the core transcriptome of *KMT2A*-R leukemia

**Fig. 6:**
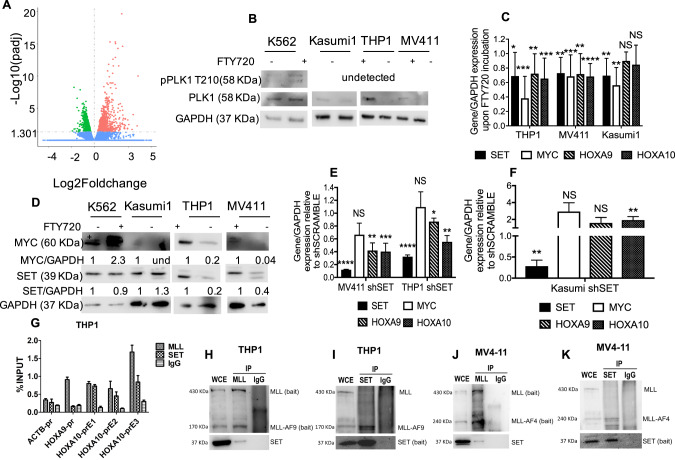
Gene expression profile analysis of FTY720 treated *KMT2A*-R leukemic cells. **A** Volcano plot of gene expression differences for THP1 cells treated with FTY720 for 24 h. The dots to the left of 0 represent gene probes with adjusted *p* value for multiple testings (padj) < 0.05 and fold change >1.3 (log2fold change < 0). The dots to the right of 0 represent gene probes with padj < 0.05 and fold change >1.3 (log2fold change > 0). **B** Immunoblot for phospho-pLK1 (Thr210) (58 kDa), PLK1 (58 kDa) and GAPDH (37 kDa) in K562, Kasumi1, THP1 and MV411 upon 5 µM FTY720 treatment for 24 h. Densitometry analysis was conducted by LI-COR Image Studio software. GAPDH was used as a loading control. **C** qRT-PCR showing the expression of *SET*, *MYC, HOXA9, and HOXA10* in leukemic cells upon 5 µM FTY720 treatment for 24 h. Gene expression was normalized by *GAPDH* control and analyzed by Pfaffl equation. Values are expressed relative to vehicle controls. Data represent mean ± SD of three independent experiments. Two tailed paired *t* test *p < 0.05; **p < 0.01; ***p < 0.001. **D** Immunoblot for SET and MYC in leukemic cells upon FTY720 5 µM treatment for 48 h. Densitometry analysis was conducted by LI-COR Image Studio software. GAPDH was used as a loading control. Values are expressed relative to vehicle control. **E**, **F** qRT-PCR showing the expression of *SET*, *MYC, HOXA9, and HOXA10* in leukemic cells transduced with lentiviral vectors expressing shSET and selected with puromycin for 72 h. Gene expression was normalized by *GAPDH* control and analyzed by Pfaffl equation. Values are expressed relative to shSCRAMBLE controls. Data represent mean ± SD of two independent experiments. Two tailed paired t-test ***p < 0.001; ****p < 0.0001. **G** Chromatin immunoprecipitation (ChIP) experiments were performed by using anti-MLL (KMT2A/MLL) and anti-SET (SET) antibodies (Ab); ChIP with anti-IgG (IgG) represents the negative control. ChIP data are expressed as percentage of specific target gene promoter elements (pr) (i.e., HOXA9-pr, HOXA10-prE1, HOXA10-prE2, HOXA10-prE3, ACTB-pr) in precipitated chromatin compared with the INPUT (% INPUT), where ACTB-pr (the promoter of ACTIN) was the negative control. Results represent the mean ± SD average of three independent experiments. **H**–**K** Co-immunoprecipitation experiments were performed using whole cell extracts (WCE), anti-MLL or anti-SET antibodies (Ab). MLL, MLL-AF9 and MLL-AF4 proteins were immunoprecipitated as baits with the anti-MLL Ab (IP MLL) in the respective cell lines and the presence of SET was revealed by western blot (WB) (left panels); SET was immunoprecipitated as bait (IP SET) with the anti-SET Ab and the presence of wild type MLL or MLL-AF9 or MLL-AF4 was analyzed by WB with the anti-MLL Ab (right panels). Immunoprecipitation with anti-IgG (IP IgG) was used as negative control.

As the phospho-proteomics indicated phosphorylation changes in targets involved in transcription (Fig. 5C), we performed RNA-seq analysis of *KMT2A*-R cell line THP1 treated with FTY720 for 24 h, to capture early changes in genetic expression, preceding cell cycle arrest and apoptosis driven by FTY720 over 48–72 h. Volcano plot filtering was used to identify differentially expressed genes between vehicle control group and FTY720 -treated group (Fig. 6A). 980 genes were significantly upregulated and 898 genes were significantly downregulated by FTY720 (fold change >1.3, *p*_adj_ < 0.05). Functional annotation clustering of these differentially expressed genes using the Gene ontology (GO), Kyoto Encyclopedia of gene and genomes (KEGG) and Reactome annotation databases indicated that sets of down-regulated genes were highly enriched in functional groups that related to ribosome biogenesis and rRNA processing (Supplementary Fig. 8A), whereas up-regulated genes were highly enriched in functional groups that related to myeloid cell activation (Supplementary Fig. 8B). In addition, upon treatment with FTY720, genes involved in autophagy, intrinsic apoptotic signaling pathway, extrinsic apoptotic signaling pathway and cell cycle arrest resulted upregulated (Supplementary Fig. 8B). Thus, these data corroborate our previous findings on cell cycle arrest and apoptosis and also indicate that FTY720 can induce cell death by multiple mechanisms, as reported in other models [44, 45, 50, 51]. To investigate whether there was any overlap between the phospho-proteomic data and the RNA-seq data, we intersected the two datasets. The results indicate that 59 out of 980 genes upregulated by FTY720, code for proteins that undergo changes in phosphorylation and 98 out of 898 genes downregulated by FTY720, code for protein that undergo changes in phosphorylation (Supplementary Fig. 8C, D and Supplementary Tables 4 and 5). Among the genes down-regulated by FTY720 and that encode for proteins that are also hypo-phosporylated by FTY720, we validated PLK1, a downstream target of Aurora kinase B and mediator of MYC stability [49, 52, 53] (Fig. 6B). The western blot analysis indicates that PLK1 is downregulated in Kasumi1, THP1, and MV411 upon FTY720 treatment (Fig. 6B). Phospho-PLK1 (T210), a phosphosite regulated by AURB, which was identified in our phospho-proteomics, was undetectable in these cell lines. In K562, PLK1 expression and phosphorylation on T210 did not change upon treatment with FTY720 or when SET was KD (Supplementary Fig. 9A). More importantly the RNA-seq data indicated that, upon FTY720 treatment, several genes overexpressed in cancer, including *MYC* and *SET* were downregulated (Supplementary Table 6), as well as genes associated with histone methyltransferase activity, among which several members of the *KMT2A*- fusion epigenetic complex, and *HOXA9/MEIS1* target genes [3, 8–10] (Supplementary Table 7). We performed RT-qPCR and western blot to validate the decreased expression of *MYC* and *SET* upon FTY720 treatment (Fig. 6C, D and Supplementary Fig. 9B-C). Upon FTY720 treatment, *MYC* mRNA and protein expression was down-regulated in Kasumi1 and in the *KMT2A*-R cell lines, but not in K562 (Fig. 6C, D and Supplementary Fig. 9B-C). *SET* mRNA was down-regulated in all leukemic cells (Fig. 6C) but, SET protein was significantly reduced only in *KMT2A*-R-cells (Fig. 6D and Supplementary Fig. 9B). RT-qPCR also confirmed a specific decrease in the expression of the *KMT2A* target genes *HOXA9* and *HOXA10*, in *KMT2A*-R-cells, upon FTY720 treatment (Fig. 6C); these genes were also specifically down-regulated in *KMT2A*-R-cells, but not in Kasumi1, when SET was knocked down (Fig. 6E, F), suggesting that SET might regulate the expression of *HOXA* genes in *KMT2A*-R-cells. Previous reports indicated that SET and the oncofusion protein SET::NUP214 modulate the expression of *HOXA* gene cluster in HeLa cell line and in T-ALL primary samples [54–56]. To identify whether SET was enriched on *HOXA9* and *HOXA10* promoters, we performed chromatin immunoprecipitation (ChIP) experiments. As expected, KMT2A localized on the promoters of both *HOXA9* and *HOXA10*; in contrast, SET was enriched only on *HOXA10* promoter (Fig. 6G and Supplementary Figs. 9E and 10). Immunoprecipitation experiments revealed that SET interacted with both KMT2A and KMT2A-fusion protein (Fig. 6H–K and Supplementary Fig. 9F–G). Collectively, our results indicate that genetic and pharmacological modulation of SET rewires the *KMT2A*-R- core gene expression signature and reduces the expression of key genes critical for sustaining this disease.

### FTY720 leads to increased sensitivity of *KMT2A*-R leukemia to chemotherapy

**Fig. 7:**
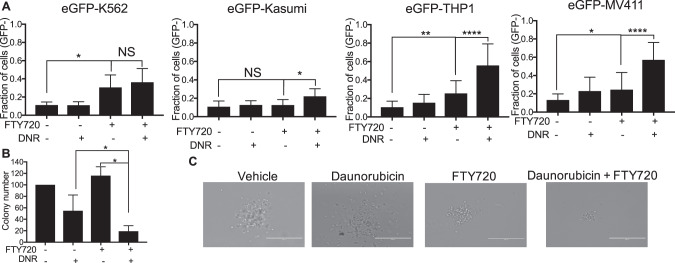
FTY720 increases the response to Daunorubicin in *KMT2A*-R leukemic cells and PDX. **A** Analysis of cell death. Cells were treated with 5 µM FTY720, 10 nM Daunorubicin or combination for 72 h. GFP signal was used as quantitative reporter of alive, non-dead cells and measured by FACS. Data show mean ± SD of triplicate wells and are representative of three independent experiments. 2-Way Anova Tukey’s multiple comparison test *p < 0.05; **p < 0.01; ***p < 0.001; ****p < 0.0001. **B** Effect of 5 µM FTY720, 10 nM Daunorubicin or combination on colony-Forming Unit ability of *KMT2A*-PDX samples. Data show the percentage of colonies in comparison to vehicle treated cells and the mean ± SD of duplicate wells and are representative of two independent samples ** *p* < 0.01. **C** Colony morphology of *KMT2A-*R-PDX. Cells were treated with the drugs in methocult for 14 days. Digital microscope images were captured using Evos FL Digital Inverted Fluorescence Microscope (magnification 40×).

Daunorubicin is an anthracycline included in the intensive multiagent chemotherapy to induce remission in *KMT2A*-R-AML patients [1]. We investigated whether SET targeting via FTY720 could enhance daunorubicin-induced cytotoxicity in *KMT2A*-R-cells. To this aim, we tested 5 µM FTY720 with 10 nM daunorubicin, a concentration that had shown a very modest effect on the proliferation and survival of AML cells, including those carrying *KMT2A-*translocations. Whereas the combination treatment FTY720 + Daunorubicin did not have any effect on eGFP-K562, the percentage of dead cells was significantly high for all the AML cell lines (Fig. 7A). The cell death increase was modest only for eGFP-Kasumi1 cell line, but the effect was very strong in *KMT2A*-R-cell lines, namely eGFP-THP1 and eGFP-MV411 (Fig. 7A and Supplementary Fig. 11). To assess the biological importance and therapeutic relevance of SET targeting via FTY720 in *KMT2A*-R-leukemia, we tested FTY720 in combination with daunorubicin in two *KMT2A*-R-patient-derived-xenograft (PDX) models in vitro. Combination treatment resulted in a significant reduction of colony number compared with vehicle and with single drug treatment of all samples (74% reduction in comparison to vehicle) (Fig. 7B, C), suggesting that FTY720 treatment enhances the response to daunorubicin.

## Discussion

**Fig. 8:**
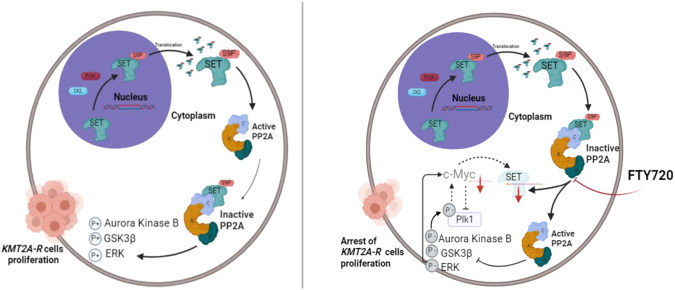
Molecular mechanisms underlying FTY720 effects in KMT2A-R-leukemic cells. Schematic cartoon representing the molecular mechanism underlining the effect of FTY720 in *KMT2A*-R- leukemic cells. The survival and proliferation of KMT2A-R leukemic cells is maintained by the activation of signaling pathways regulated by multiple kinases including ERK1, GSK3β and AURB (left). FTY720 disrupts the binding between SET and PP2A, re-activating PP2A, that leads to inactivation of multiple kinases (right). The restored activity of PP2A affects MYC transcription via AURB, and MYC protein stability, via ERK1, GSK3β and PLK1. The latter is also a downstream effector of AURB. The overall effect is suppression of MYC transcription and MYC stability. This, in turn, affects the transcription of genes regulated by MYC, including PLK1 and SET. The inhibition of SET feeds into the activation of PP2A, with profound effects on survival and proliferation of KMT2A-R-leukemic cells.

SET is an endogenous PP2A inhibitor overexpressed in several types of solid tumors and hematological malignancies [21–26]. Mechanistically, SET over-expression and the resulting PP2A inhibition is critical for the maintenance of the leukemogenic program by BCR::ABL in CML [21, 27]. In contrast, the exact molecular mechanisms linking a SET oncoprotein pathway with aggressive AML outcomes are not known. Here we report that SET is over-expressed in the *KMT2A*-R subtypes of AML and ALL and it positively correlates with the expression of *MEIS* and *HOXA* genes. We also show that, in *KMT2A*-R-cell lines, SET is relatively more abundant in the cytoplasm than in the nucleus and is phosphorylated on Serine residues, which is in line with the notion that the nuclear import of SET is inhibited by phosphorylation on SER9 and SER24 [26, 33, 34]. We demonstrate that SET silencing has a detrimental effect on the proliferation of *KMT2A*-wt- leukemic cells, as also observed in CML and solid tumors [21, 57–59]. More importantly, we show for the first time that SET silencing completely abolishes the ability of *KMT2A*-R-leukemic cells to form colonies in semi-solid medium, indicating that SET plays an important role in the clonogenic ability of *KMT2A*-R-cells. To provide a proof of concept that pharmacological modulation of SET is a promising strategy for the treatment of *KMT2A*-R-leukemia, we tested in vitro FTY720 (Fingolimod), a proven inhibitor of the interaction between SET and PP2A [28, 40–43], that had been shown to induce cell death in several solid tumors and leukemia models by various mechanisms [44, 45, 50, 51]. In agreement with previous reports on CML [28], we show that FTY720 has a modest negative impact on the proliferation of *BCR:ABL* + *KMT2A*-wt- leukemic cell line K562 and promotes cell death. In *KMT2A*-wt- leukemic cell line REH, FTY720 has a greater cytostatic and cytotoxic impact, in agreement with data previously published [44]. In contrast to [45], in Kasumi1, FTY720 has a modest effect on proliferation and it does not induce cell death. Whereas both FTY720 and SET KD have a similar impact on *KMT2A*-wt cell lines K562 and REH, FTY720 does not fully mimic the effect of SET KD in Kasumi1, as these cells are more sensitive to SET KD than to FTY720 treatment. This might be explained by the residual level of SET. Indeed, whereas the KD induces a 90% reduction in SET protein levels, FTY720 does not reduce the expression of SET protein in Kasumi1. In *KMT2A*-R cells, FTY720 causes cell cycle arrest and promotes cell death. Accordingly, our phospho-proteomic and RNA-seq analyses in KMT2A-R-models reveal decreased activity of kinases implicated in cell division and enhanced expression of genes promoting cell cycle arrest and apoptosis. In particular, phospho-proteomic data show that FTY720 has an inhibitory effect on the signaling mediated by AURB, PLK1, ERK1 and MYC, suggesting reactivation of their upstream negative regulator PP2A [47]. Rescue experiments with the PP2A inhibitor okadaic acid, as well as by silencing of *PPP2CA*, the gene encoding for PP2A catalytic subunit α, prove that some of the effects of FTY720 on *KMT2A*-R-cells are dependent on PP2A activation. We are aware that some of the proteomic changes observed might not be due to PP2A activation or they might be compensatory effects. Further studies in PPP2CA and PPP2R1A KD cells are needed to validate PP2A as mediator of each of these aforementioned signaling pathways. Significantly, phospho-proteomic analyses indicate that FTY720 affects pathways implicated in gene transcription. RNA-seq analysis shows that FTY720 reduces the expression of SET mRNA, a result confirmed by RT-qPCR in *KMT2A*-R as well as *KMT2A*-wt-leukemic cells. Interestingly, western blot experiments show a sustained and specific reduction of SET protein expression following FTY720 treatment only in *KMT2A*-R-leukemic cells. This latter result uncovers a novel molecular mechanism underlying FTY720 action, distinct from the current understanding of FTY720 as a sphingosine mimetic capable of disrupting the interaction between SET and PP2A without affecting the levels of SET [41, 42, 46]. The effect of FTY720 on SET transcription could be explained by the modulation of MYC, which is a transcriptional activator of SET [60]. Previous studies recognized MYC as a critical substrate of PP2A complex in cancer [58, 59, 61]. In pancreatic and breast cancer cells, PP2A activation by KD of SET or by the SET antagonist OP449 decreases Ser62 and Thr58 phosphorylation of MYC and directs MYC towards ubiquitin-mediated proteasomal degradation [58, 59]. Our data support the hypothesis of a feedforward loop between PP2A, AURB, PLK1, MYC, and SET whereby FTY720 reduces MYC transcription, by reducing the activity of AURB, an upstream regulator of MYC [49], and it compromises MYC stability, by reducing the activity of AURB, PLK1, ERK1 and GSK3β, which modulate MYC by phosphorylation on S62 and T58 [49, 52, 53, 61]. Proteasomal degradation of MYC suppresses MYC-dependent gene transcription and therefore expression of downstream targets PLK1 [53] and SET [60] which, in turn, might feed into the activation of PP2A, with profound effects on survival and proliferation of leukemic cells (Fig. 8). What our model does not fully explain is how SET KD or pharmacological inhibition by FTY720 regulate the expression of *KMT2A* signature genes. Our data indicate that SET interacts with both KMT2A wt and KMT2A fusion proteins and that it is recruited to the promoter of *HOXA10*, suggesting that SET might recruit KMT2A to the promoter of this gene or vice versa. This is supported by a yeast two hybrid screening that reported KMT2A as a SET interactor [29] and by experiments performed in HeLa cells showing that the interaction between SET and KMT2A has a synergistic effect on the activation of *HOXA* gene expression [54]. More recent studies report that the oncofusion protein SET::NUP214, which has been found in a subset of T-ALL patients with abnormal expression of *HOXA* gene cluster, recruits both KMT2A and DOT1L to the promotor regions of *HOXA9* and *HOXA10* [55, 56]. Notably, a phospho-proteomic study in HeLa cells, where *SET* was knocked down by RNAi, identified several targets directly involved in RNA Polymerase II (RNAPII)-mediated transcription and RNA processing, providing a possible link between SET, PP2A, and gene transcription [62]. In addition, a study identified CDK9, an important regulator of RNAPII and critical player in KMT2A-fusion-mediated transcription [18], as a PP2A target, corroborating the crosstalk between phosphorylation and transcription [63]. Moreover, in a recent study, AAkula et al. combined the data from two phospho-proteome studies, including the one performed on HeLa where SET was downregulated by siRNA [62], to identify phospho-proteins co-regulated by RAS and PP2A [64]. The study indicates that PP2A- and RAS-mediated phosphorylation converge on epigenetic complexes, including the DOT1L complex. The DOT1L phosphosites Ser900 and Ser1104, identified as dephosphorylated by siSET in the study by Aaakula et al., were also found dephosphorylated by FTY720 in THP1 (Supplementary Table 2), supporting the concept that SET mediates its anti-tumor effects though modulation of relevant epigenetic factors. Another SET-dependent DOT1L phosphosite identified by Aakula et al., Ser1001, was found to correlate positively with *HOXA* gene expression in *KMT2A*-R-AML patients [65]. Therefore, the molecular mechanisms implicated in the SET-mediated regulation of KMT2A transcriptional signature warrant further investigation. Integrating these published phospho-proteomes and validating the functional role of some of these phosphorylation sites might might reveal further mechanistic insights into the role of SET and PP2A inhibition in leukemic transcription and chromatin accessibility and should be explored in future studies.

Consistent with its anti-proliferative effect, FTY720 treatment was shown to be synergistic with cytotoxic chemotherapeutic agent, doxorubicin, in solid tumors [66, 67]. We demonstrated that FTY720 increases the response of *KMT2A*-R-cells to daunorubicin, a standard chemotherapeutic agent used in induction and consolidation treatment for *KMT2A*-R patients. This effect might be partially dependent on the impact of FTY720 on the DNA damage response barrier; by hyperactivating error-prone DDR pathways, such as the NHEJ via PRKDC (DNA-PK), and by simultaneously inhibiting HR-mediated DNA repair via modulation of BRCA1 phosphorylation, FTY720 might offset the DDR HR- mediated, essential to repair faithfully the DNA damage lesions induced by daunorubicin, and drive the leukemic cells to death.

Since the anti-cancer effect of FTY720 is an off-target effect and the dose used as an anticancer drug is much higher than the dose used as immune-suppressor, it has been postulated that FTY720 might be too toxic to be used in clinics. In THP1-based mouse models, we observed immunosuppression, lymphopenia and severe weight loss (data not shown). As FTY720 exerts its immunosuppressive properties after being phosphorylated in vivo in FTY720-P, a mimetic of sphingosine-1-phosphate (S1P) critical for lymphocyte trafficking [40], and several studies indicate that the phosphorylation of FTY720 is not essential to exert anti-leukemic effects [27, 28, 50], studies have been focused on the development of FTY720 analogs with anti-cancer but no immunosuppressive properties. Such molecules (AAl-149 (S), (S)-FTY720-OMe, (S)-FTY720-regioisomer, and OSU- 2S, MP07-66, CM-1231) have been shown to activate PP2A and induce apoptosis in leukemia models [46, 68, 69]. Furthermore, SH-RF-177, a FTY720 analog that is efficiently phosphorylated but that does not activate S1P receptor 1, shows anti-leukemic activity on ALL cells [70]. Therefore, further studies are needed to evaluate the potential of these analogs in *KMT2A*-R-leukemia.

In conclusion, our study demonstrates for the first time that the expression of PP2A inhibitor SET positively correlates with *MEIS* and *HOXA* genes. SET is critical for the clonogenic ability of *KMT2A-*R-leukemic cells and can be pharmacologically targeted by using FTY720, an immunosuppressive sphingosine mimetic that reactivates PP2A by suppressing SET. We provide the evidence that silencing and pharmacological inhibition of SET in *KMT2A*-R-leukemic cells promote cell cycle arrest and apoptosis, rewire the transcriptional program, down-regulate the expression of *HOXA* genes and direct MYC toward proteasomal degradation. These data shed light on SET as a new therapeutic target of *KMT2A*-R leukemia.

## Material and methods

### Cell lines

The cells lines used for this study and the experimental conditions are indicated in supplementary material and methods. Some of the cell lines used in this study were stably transduced with a lentivirus vector expressing the enhanced green fluorescent protein (eGFP) and positive clones were sorted by FACS, as described [38]. The cell lines eGFP-K562, eGFP-Kasumi1, eGFP-THP1, eGFP-MV411, eGFP-REH, eGFP-SEM, eGFP-Hb1119, and eGFP-U937 were further tested for authenticity by STR profiling (Eurofin Genomics).

### Primary cells

Primary samples, described in supplementary material and methods, were obtained from the Cancer Tissue Bank at the Barts Cancer Institute (London) under ethical approval (REC reference: 17/WM/0428).

### PDX samples

The KMT2A-PDX, described in supplementary material and methods and in [71, 72], were a generous gift of Prof. Owen Williams.

### Virus production and cell transduction

Knock-down of *SET* was conducted in vitro using the lentiviral viruses purchased from Sigma-Merck, as described in supplementary material and methods. The viruses are based on the plasmid vector pLKO.p1 co-expressing a puromycin resistance cassette to enable transduction selection of mammalian cells and ensure the establishment of stable clones. As a transduction negative control, we used non targeting shRNA control (shScramble), designed to target no known gene sequence. The pLKO, 1-puro CMV-tag red fluorescent protein (RFP), with no shRNA insert and expressing the RFP, was used as a transduction positive control.

### Cell proliferation and cell death analysis

Cell proliferation and cell death were monitored by GFP fluorescence and by measuring the percentage of GFP negative cells as described in ref. [38] and in supplementary material and methods.

### Reagents

FTY720 was purchased from Selleckchem (S5002). The antibodies used in this study are listed in supplementary material and methods.

### RT-qPCR and ChiP

Quantitative real-time PCR (RT-qPCR) was performed using specific primers from Sigma-Merck. ChIP assay was performed as previously reported [6, 73]. The primers are listed in supplementary material and methods.

### Phospho-proteomic experiments

Phospho-proteomic experiments were performed using mass spectrometry as reported in refs. [48, 74] and in supplementary material and methods.

### Microarray, RNA-seq and bioinformatic analysis

The expression profile of *SET* in human HSC and blood cells was obtained from Bloodspot Gene expression profiles (GSE42519) [31].

The expression profile of *SET mRNA* in AML patients was obtained from Leucegene gene expression profiles (GSE62190, GSE66917, GSE67039) [10].

The expression of mouse *SET* mRNA (probe1421819_a_at) was analyzed by using the micro-array data from GSE13690 [35]. This dataset was generated by Somervaille et al., by analyzing the transcriptional profiles of 34 mouse *KMT2A*-R-AML. In this study, Somervaille et al. assessed the relative LSC frequency of these AML by determining the colony-forming cell (CFC) frequencies of spleen and bone marrow cells collected from leukemic mice. The transcriptional profiles were split in two groups: a group labeled as high-LSC frequency (comprising AML initiated by *KMT2A::MLLT3* and *KMT2A:MLLT1*) and one labeled as low-LSC frequency (comprising AML initiated by *KMT2A::AFF1p*, *KMT2A::AF10* and *KMT2A::GAS7*).

Meta-analyses of micro-array data from the PrognoScan database (GSE12417) were used to determine the prognosis of AML patients expressing high or low levels of SET [32]. The data represent the analysis of 163 patients treated in the German AMLCG 1999 trial. The survival analysis in PrognoScan is based the minimum p-value approach to find the cutpoint in continuous gene expression measurement for grouping patients.

The publicly accessible online database cBioPortal (https://www.cbioportal.org/, accessed on 29 May 2023) was used to explore the correlation of SET with the self-renewal leukemic stem cell (LSC) marker genes published by Gentles et al. [36] and by Krivtsov et al. [37]. We checked four AML cohorts: OHSU (Cancer cell 2022, *n* = 942), OHSU (Nature 2018, *n* = 672), TCGA (Firehose Legacy, *n* = 200) and TCGA (NEJM 2013, *n* = 200). The co-expression of SET with the genes of interest was tested by the Spearman’s correlation coefficient (Rs), that summarize the strength and direction (positive or negative) of a relationship between two variables. A significant correlation of co-expression with SET was considered according to the absolute value of the Spearman’s correlation coefficient as weak (0.20–0.39), moderate (0.40–0.59), strong (0.60–0.79) or very strong (0.80–1) when the *p* value was lower than 0.05 (*p* < 0.05). The graphs were made using GraphPad Prism 9 software.

The RNA-seq in FTY720- vs vehicle-treated THP1 cells was performed by Novogene. Downstream analysis was performed using a combination of programs including STAR, HTseq, Cufflink and Novogene wrapped scripts. Differential expression analysis between two conditions/groups (three biological replicates per condition) was performed using the DESeq2 R package (2_1.6.3), as described in supplementary material and methods.

### Statistical analysis

Data are expressed as mean ± standard deviation (SD) from at least three independent experiments, unless stated otherwise. Statistical significance was determined using GraphPad Prism 7 with the tests reported in the figure legends.

Additional methods are available in the supplementary materials and methods.

### Supplementary information

supplementary data and material and methods

Supplementary table 1

Supplementary table 3

Supplementary table 4

Supplementary table 5

Supplementary Figure 1

Supplementary Figure 2

Supplementary Figure 3

Supplementary Figure 4

Supplementary figure 5

Supplementary Figure 6

Supplementary Figure 7

Supplementary Figure 8

Supplementary Figure 9

Supplementary Figure 10

Supplementary Figure 11

## Data Availability

The FTY720-RNASeq datasets generated during the current study are available in the GEO repository (GSE218708). The three plot shown in Fig. 1 has been drawn based on data obtained from Bloodspot Gene expression profiles (GSE42519). The heatmap shown in Fig. 1 has been generated by analyzing data available from Leucegene (GSE62190, GSE66917, GSE67039). The expression of SET mRNA in murine KMT2A-LSC has been obtained by meta-analyses of micro-array data from GSE13690. Kaplan–Meier analysis of AML patients with high and low expression levels of SET is based on micro-array data from the PrognoScan database (GSE12417). The mass spectrometry proteomics data have been deposited to the ProteomeXchange Consortium via the PRIDE partner repository with the dataset identifier PXD038288 and 10.6019/PXD038288.
