# Supplementary material for: SET-PP2A complex as a new therapeutic target in KMT2A (MLL) rearranged AML
Source: Oncogene. 2023 Oct 27;42(50):3670–83. doi: 10.1038/s41388-023-02840-1 (PMC10709139; doi:10.1038/s41388-023-02840-1)
Supplement: Supplementary file 1 — supplementary data and material and methods [file 41388_2023_2840_MOESM1_ESM.docx]

**Supplementary figure legends**

**Supplementary Figure 1**

**A)** Micro-array gene expression profile of *SET mRNA* in human leukemia, HSCs, progenitors and differentiated blood cells. Data obtained from Bloodspot gene expression profiles GSE42519 (n=34) and GSE13159 (n=542); AML with t(8;21) n=40, AML with t(15;17) n=37, AML with inv(16)/t(16;16) n=28, AML with t(11q23)*MLL* n=38, AML with normal karyotype n=351, AML complex aberrant phenotype n=48, hematopoietic stem cells (HSC) n=4, multipotential progenitors (MPP) n=2, common myeloid progenitors (CMP) n=3, granulocyte myeloid progenitors (GMP) n=5, megakaryocyte-erythroid progenitor cells (MEP) n=2, early promyelocyte (ea_PM) n=3, late promyelocyte (late_PM) n=3, myelocyte (MY) n=2, metamyelocyte (MM) n=3, band cell (BC) n=4, Polymorphonuclear cells n=3. **B)** and **C)** RT-qPCR showing the expression of *SET mRNA* in the mononuclear cells isolated from the bone marrow of healthy volunteers (BM), KMT2A-R-leukemic patients and in a range of AML and ALL human cell lines. Gene expression was normalized by *GAPDH* control and analysed by Pfaffl equation. Values are expressed relative to BM. Data represent mean +/- SD of three independent experiments. 2-Way Anova Dunet’s multiple comparison test *p<0.05;** p<0.01;*** p<0.001. **D), E)** and **F)** Protein complex -immunoprecipitation showing SET phosphorylation interaction in leukemic cells. Immunoblotting against SET (39KDa) was performed after Immunoprecipitation with a phosphoserine antibody (FT: Flow through control; IgG HC: IgG high chains; IgG LC: IgG low chains).

**Supplementary Figure 2**

**A)** Colony morphology of K562 (*KMT2A*-wt, *BCR::ABL* t(19;22) erythroleukemia cell line), Kasumi1 (*KMT2A*-wt *AML1::ETO* t(8;21) AML cell line), THP1 and MV411 (*KMT2A*-R- AML cell lines), REH (KMT2A-wt *TEL::AML1* t(12;21) ALL), HB119 and SEM1 (*KMT2A*-R-ALL) upon lentiviral transduction with vectors expressing shSCRAMBLE, RFP or shSET under puromycin selection. Digital microscope images were captured using Evos FL Digital Inverted Fluorescence Microscope (magnification 40X). Data are representative of three independent experiments. **B)** Leukemic cells were infected with a lentiviral vector expressing eGFP and positive cells have been isolated by cell sorting. The fluorescence emission is proportional to the number of cells. Representative FACS plots indicating the Forward and Side scatter of THP1 cells included in the analysis (population P1), the gating strategy to exclude duplets from the analysis (population P2) and the gates M1 and M2 to discriminate GFP- from GFP+ cells. **C)** Digital microscope images of eGFP-leukemic cell lines untransduced and transduced with lentiviral vector pLKO.1 expressing RFP 48 hours after transduction. The RFP signal was used as a reporter of transduction efficiency. Untransduced cells exhibited only GFP emission. Successfully transduced cells exhibited both GFP and RFP emission. Images were captured using Evos FL Digital Inverted Fluorescence Microscope (magnification 20X). **D)** Giemsa-Wright staining of cells isolated from colonies shown in Figure 3. Images were taken using Nikon Digital camera adapter for microscope (D5100) (magnification 20X). Data are representative of a single experiment.

**Supplementary Figure 3**

**A-C)** Proliferation curve of eGFP-K562 and eGFP-Kasumi1 and eGFP-REH, stably expressing shScramble, or shSET, determined by MTS assay. The graphs reported the optical density (OD) measured at 490 nm. Data show mean +/-SD of triplicate wells and are representative of three independent experiments. 2-Way Anova Sydak’s multiple comparison test *p<0.05; **** p<0.0001. **D-O)** Pearson’s correlation between the results of the proliferation assay determined by GFP monitoring and by MTS assay. The graphs report the R square for each correlation. **P-V)** Proliferation curve of eGFP-K562 and eGFP-Kasumi1, eGFP-THP1, eGFP-MV411, eGFP-REH, eGFP-Hb1119 and eGFP-SEM, treated with 5 μM FTY720 and determined by MTS assay. The graphs reported the optical density (OD) measured at 490 nm. Data show mean +/-SD of triplicate wells and represent three independent experiments. 2-Way Anova Sydak’s multiple comparison test *p<0.05; ** p<0.01; *** p<0.001; **** p<0.0001.

**Supplementary Figure 4**

**A)** Non-linear regression dose/ response curve of FTY720 treatment in leukemic cell lines. Cell viability was determined by Trypan blue exclusion upon FTY720 treatment for 72hrs. The FTY720 IC50 was calculated using GraphPad Prism software. Data shown as average +/-SDof triplicate wells and are representative of three independent experiments. **B)** Flow cytometry histograms representing analysis of cell cycle by propidium iodide staining upon 5 µM FTY720 treatment for 48 hrs. **C)** Flow cytometry histograms representing analysis of cell death upon FTY720 treatment. Cells were treated with FTY720 5µM for 72 hours. GFP fluorescent signal was used as quantitative reporter of alive cells and measured by flow cytometry. The gates M1 and M2 discriminate GFP- from GFP+ cells.

**Supplementary Figure 5**

**A)** Proliferation curve of eGFP-K562 upon treatment with 2.5 nM and 5 nM Okadaic Acid for 3 days. The GFP was used as quantitative reporter of cell proliferation. For each condition, the same number of cells was plated at time 0 and the GFP signal was measured with a fluorescent microplate reader. Data show mean +/-SD of triplicate wells and are representative of three independent experiments. **B**)Immunoblot for phosphoAKT1/2 (Ser473) (60KDa), AKT (pan) (60KDa), phosphoGSK3β (Ser9) (46KDa), GSK3β (46KDa), phosphoERK1/2 (Thr402/Tyr404) (42-44KDa), ERK1/2 (42-44KDa), and GAPDH (37KDa), in K562 upon 2.5nM and 5nM Okadaic Acid treatment for 2-4 and 6 hours. Densitometry analysis was conducted by LI-COR Image Studio software. GAPDH was used as a loading control. **C**)Immunoblot for phosphoAKT1/2 (Thr308) (60KDa), AKT (pan) (60KDa) and GAPDH (37KDa) in K562 upon 2.5nM and 5nM Okadaic Acid treatment for 2-4 and 6 hours. Densitometry analysis was conducted by LI-COR Image Studio software. GAPDH was used as a loading control.

**Supplementary Figure 6**

Flow cytometry histograms representing analysis of cell death of upon FTY720 and Okadaic Acid (OA) treatment. Cells were treated with 2.5nM Okadaic Acid and 5µM FTY720 for 72 hours. GFP fluorescent signal was used as quantitative reporter of alive cells and measured by flow cytometry. The gates M1 and M2 were used to discriminate GFP- from GFP+ cells.

**Supplementary Figure 7**

Phospho-proteomic analysis of eGFP-THP1 and eGFP-MV411 cells treated with FTY720. **A)** and **B)** Principal component analysis distinguishes cells treated with 5 μM FTY720 for 48 hours from cells treated with vehicle. **C)** and **D)** Correlation analysis. Spearman rank correlation coefficients for all pairwise sample comparisons. **E)** and **F)** Phosphosite clustering and Gene Ontology Biological Process analysis in eGFP-THP1 and eGFP-MV411 cells treated with FTY720 for 48 hours. Upon treatment with 5 μM FTY720 for 48 hours eGFP-THP1 cells phosphorylation profile was impacted, with 2276 phosphosites significantly altered >2 fold and p<0.1 and 1862 phosphosites significant >2 fold and <0.01. Upon treatment with 5 uM FTY720 for 48 hours eGFP-MV411 cells phosphorylation profile was impacted, with 1428 phosphosites sig at >2 fold and <0.1 and 743 phosphosites significant >2 fold and <0.01. (a) Shows the results of clustering these 2276 phsophosites for eGFP-THP1 and (b) shows the results of clustering 1428 phosphosites from eGFP-MV411 cells. Abundances were z-transformed by row. Euclidean distance average linkage clustering was then applied to these datapoints and clustered heatmaps shown. Each clustered set of phosphoproteins was analysed with Gene Ontology and the representative terms annotated onto each cluster. **G)** to **K)** Hierarchical clustering of phosphosites allocated to specific kinases by KSEA analysis. To create groups of phosphorylation sites that share similar patterns of abundance changes targets of specific kinases were grouped together by KSEA analysis. Log2 fold-change of >1 and p-value <0.1 filters were applied to exclude phosphosites that were unchanged in abundance and phosphosite abundances were z-transformed by row. Euclidean distance average linkage clustering was then applied to these datapoints and clustered heatmaps shown.

**Supplementary Figure 8**

RNA-seq results identify differentially regulated genes in THP1 cell treated with FTY720 for 24 hours. **A)** Gene Ontology. Genes upregulated in FTY720 treated cells at fold change >1.3, padj<0.05 cut-offs. **B)** Gene Ontology. Genes downregulated in FTY720 treated cells at fold change >1.3, padj<0.05 cut-offs. **C)** and **D)** Venn diagram showing comparison between RNAseq data and phosphoproteomics data. Cutoff for RNAseq data was set to fold change >1.3, padj<0.05; the cutoff for the phosphoproteomics data was set to fold change>2 fold , p<0.01).

**Supplementary Figure 9**

**A)** Immunoblot for SET (39KDa), phospho-MYC Ser62 (60KDa), phospho-MYC Thr58 (60KDa), MYC (60KDa), phosphoPLK1 (Thr210) (58KDa), PLK1 (58 KDa) and GAPDH (37KDa) in K562 expressing either shScramble or shSET. Densitometry analysis was conducted by LI-COR Image Studio software. GAPDH was used as a loading control. **B)** Immunoblot for SET (39KDa), MYC (60KDa), phosphor-MYC Ser62 (60KDa) and GAPDH (37KDa) in eGFP-K562 upon FTY720 5uM treatment for 24 and 48hours. Densitometry analysis was conducted by LI-COR Image Studio software. GAPDH was used as a loading control. Values are expressed relative to vehicle control. **C)** and **D)** qRT-PCR showing the expression of *SET* and *MYC* upon 5 µM FTY720 treatment for 24hrs (C) and in K562 expressing either shScramble or shSET (D). Gene expression was normalized by *GAPDH* control and analysed by Pfaffl equation. Values are expressed relative to vehicle controls. Data represent mean +/- SD of three independent experiments. Two tailed paired t test ** p<0.01. **E)** Chromatin immunoprecipitation (ChIP) experiments were performed by using anti-MLL (KMT2A/MLL) and anti-SET (SET) antibodies (Ab); ChIP with anti-IgG (IgG) represents the negative control. ChIP data are expressed as percentage of specific target gene promoter elements (pr) (i.e. HOXA9-pr, HOXA10-prE1, HOXA10-prE2, HOXA10-prE3, ACTB-pr) in precipitated chromatin compared with the INPUT (% INPUT), where ACTB-pr (the promoter of ACTIN) was the negative control. Results represent the average of three independent experiments; error bars indicate the standard deviations. **F)** and **G)** Co-immunoprecipitation experiments were performed using whole cell extracts (WCE), anti-MLL or anti-SET antibodies (Ab). MLL protein was immunoprecipitated as baits with the anti-MLL Ab (IP MLL) in the respective cell lines and the presence of SET was revealed by western blot (WB) (left panels); SET was immunoprecipitated as bait (IP SET) with the anti-SET Ab and the presence of wild type MLL was analyzed by WB with the anti-MLL Ab (right panels). Immunoprecipitation with anti-IgG (IP IgG) was used as negative control.

**Supplementary Figure 10**

The three different *HOXA10* promoter elements are indicated as prE1, prE2 and prE3. The graphs’ histograms show the % INPUT values (as average of three independent experiments and error bars indicating the standard deviations) already reported in Figure 6 and supplementary Figure 9.

**Supplementary Figure 11**

Flow cytometry histograms representing analysis of cell death of upon FTY720 and Daunorubicin treatment. Cells were treated with 10nM Daunorubicin and 5µM FTY720 for 72 hours. GFP fluorescent signal was used as quantitative reporter of alive cells and measured by flow cytometry. The gates M1 and M2 were used to discriminate GFP- from GFP+ cells.

**List of supplementary tables:**

Supplementary table 1: Prognoscan database-based Kaplan Meier analysis of overall survival (excel file)

Supplementary Table 2: Fold increase in proliferation over 6 days (in this document)

Supplementary table 3: List of identified proteins and phosphopetides (Phospho-proteomics) (excel file)

Supplementary table 4: Analysis of overlap between RNA-Seq and Phospho-proteomics. Venn diagram 1 (excel file)

Supplementary table 5 Analysis of overlap between RNA-Seq and Phospho-proteomics. Venn diagram 2 (excel file)

Supplementary table 6 Cancer genes down-regulated in FTY720-treated THP1 (in this document)

Supplementary table 7 *HOXA9/Meis1* target genes downregulated in FTY720 treated THP1 (in this document)

Supplementary table 8 Characteristics of cell lines (in this document)

Supplementary table 9 Characteristics of the primary samples (in this document)

Supplementary table 10 Summary of of human bone marrow and peripheral blood mononuclear cells (in this document)

Supplementary table 11 Summary of shRNA (in this document)

Supplementary table 12. List of Primary Antibodies used for the study (in this document)

Supplementary table 13. List of Secondary Antibodies used for the study (in this document)

Supplementary table 14. List of primers used for the qPCR experiments (in this document)

Supplementary table 15. List of primers used for the ChiP experiments (in this document)

**Supplementary Table 2. Fold increase in proliferation over 6 days**

| **Cell line** | **Assay** | **untransduced** | **shScramble** | **shSET** | **Vehicle** | **FTY720** |
| --- | --- | --- | --- | --- | --- | --- |
| K562 | GFP | 4.049±0.34 | 3.59±0.19 | 3.08±0.16 | 2.49±0.59 | 1.58±0.58 |
| K562 | MTT | 4.19±0.95 | 4.28±0.33 | 3.43±0.83 | 4.88±1.54 | 1.79±1.74 |
| Kasumi | GFP | 5.34±0.69 | 5.20±0.29 | 1.12±0.07 | 4.7±1.07 | 4.01±0.63 |
| Kasumi | MTT | 4.59±1.0 | 3.86±0.03 | 3.57±0.09 | 4.58±1.00 | 3.53±0.20 |
| REH | GFP | 6.16±2.7 |  | 2.98±0.80 | 3.26±1.46 | 0.98±0.01 |
| REH | MTT | 4.46±0.81 |  | 2.79±0.47 | 2.39±0.46 | 0.72±0.07 |
| THP1 | GFP |  |  |  | 4.05±0.08 | 1.75±0.63 |
| THP1 | MTT |  |  |  | 4.80±0.47 | 1.76±0.87 |
| MV411 | GFP |  |  |  | 4.58±0.11 | 1.24±0.37 |
| MV411 | MTT |  |  |  | 4.53±0.52 | 0.92±0.13 |
| SEM | GFP |  |  |  | 4.48±0.24 | 1.66±0.23 |
| SEM | MTT |  |  |  | 4.56±0.87 | 1.76±1.05 |
| Hb1119 | GFP |  |  |  | 3.65±0.37 | 0.99±0.01 |
| HB1119 | MTT |  |  |  | 2.79±0.03 | 0.64±0.01 |

**Supplementary Table 6 Cancer genes down-regulated in FTY720-treated THP1**

| **gene_name** | **Log2 FoldChange** | **padj value** |
| --- | --- | --- |
| MYC | -0.994981586 | 0.00013212 |
| HSP90AB1 | -0.599623728 | 0.00057408 |
| MEN1 | -0.52520975 | 0.00199724 |
| SRSF3 | -0.485235732 | 0.00231311 |
| TERT | -1.177172735 | 0.00338404 |
| SRSF2 | -0.509520745 | 0.00674104 |
| SMARCB1 | -0.417069371 | 0.00699292 |
| AFF3 | -0.978662325 | 0.00782963 |
| DDX10 | -0.545969385 | 0.00867113 |
| CNBP | -0.373653234 | 0.00890844 |
| ATIC | -0.500996693 | 0.01452106 |
| NPM1 | -0.417991053 | 0.02614984 |
| EZR | -0.341689255 | 0.02948437 |
| CARS | -0.375857356 | 0.03135053 |
| HMGA1 | -0.402878487 | 0.03324904 |
| YWHAE | -0.37724889 | 0.03679825 |
| **SET** | **-0.312146973** | 0.03967922 |
| PMS1 | -0.395361526 | 0.04241133 |
| TCF3 | -0.329126504 | 0.04355486 |
| TAF15 | -0.397192416 | 0.04460986 |
| BCL11A | -0.549147557 | 0.04842762 |
| HOXA10 | -0.4423 | 0.040643 |

**Supplementary table 7** ***HOXA9/Meis1* target genes downregulated in FTY720 treated THP1**

| **gene_name** | **Log2 FoldChange** | **padj value** |
| --- | --- | --- |
| TFAP4 | -0.94723 | 3.71E-05 |
| RUVBL2 | -0.73011 | 9.83E-05 |
| ODC1 | -0.87831 | 0.000143 |
| FABP5 | -0.81414 | 0.000188 |
| RUVBL1 | -0.84759 | 0.000238 |
| MYBBP1A | -0.84227 | 0.000589 |
| BCAT1 | -0.67112 | 0.001026 |
| MRPL15 | -0.53858 | 0.001721 |
| NCL | -0.6414 | 0.001796 |
| IFRD2 | -0.76262 | 0.002881 |
| DNAJA3 | -0.53552 | 0.005854 |
| PPAN | -0.72923 | 0.00844 |
| NOLC1 | -0.65623 | 0.009561 |
| SNRPA1 | -0.5096 | 0.010346 |
| PRMT1 | -0.48086 | 0.012904 |
| NOP58 | -0.53462 | 0.013505 |
| TRIP13 | -0.46408 | 0.018356 |
| MCM3 | -0.45463 | 0.024747 |
| BID | -0.42466 | 0.027915 |
| TRMT2A | -0.41896 | 0.033221 |
| WEE1 | -0.37674 | 0.0033581 |

# **MATERIALS AND METHODS**

**Cell cultures**

**Cell lines**

The cells lines used for this study (supplementary table 8) were either purchased from DSMZ or were a gift of Prof. Eric and So (King’s College University, London), Dr Yolanda Calle (University of Roehampton), Prof. Owen Williams (University College London) and Dr David Giuliano (University of East London). K562, ML2, MOLM13, MV4-11, NOMO1, THP1, Hb11-19, KOPN8, SEM, REH, U937 cells, were grown in Roswell Park Memorial Institute medium (RPMI-1640) (Sigma) supplemented with 10% of Foetal Bovine Serum (FBS) and 100U/mL penicillin and 100 µg/mL streptomycin; Kasumi and RS4;11 cell line were grown in RPM1, 25mM HEPES-modified and supplemented with 20% of foetal bovine serum (FBS) and 100U/mL penicillin and 100 µg/mL streptomycin. All the cell lines were maintained in culture at 37^0^C in a 5% CO_2_, by routine passage every 2-3 days and regularly tested for mycoplasma contamination using PCR Mycoplasma detection kit from Abm. The cell lines were tested for authenticity by STR profiling (Eurofin Genomics).

**Primary samples**

Primary samples (supplementary table 9) were obtained from the Cancer Tissue Bank at the Barts Cancer Institute (London) under ethical approval (REC reference: 17/WM/0428). The samples were treated with RBC (Red Blood Cells) Lysis buffer (155mM NaCl, 10mM KHCO_2,_ 0.1mM EDTA, pH 7.3) for 10 minutes to eliminate any potential contaminant red cell. Following this, the pellets were washed with PBS and spun at 350 g for 5 minutes. Mononuclear cells (MNCs) from bone marrow (Cat. 7001.2) and peripheral blood (Cat. 70008.4) isolated from healthy donors were obtained from Stem Cells Technology (supplementary table 10).

**PDX samples**

The KMT2A-PDX were a generous gift of Prof. Owen Williams. The sample identified as 1547 was a AML sample carrying t(9;11) and isolated in Rotterdam (NE) (1). The sample 270418A was a AML sample carrying t(11;19) and isolated at GOSH (London, UK) (2).These cells were grown in methocult H4435 (Stem Cell Technologies) with addition of 10 ng/mL human TPO1 and human FLt3 ligand (Stem Cell technologies).

**Virus production and cell transduction**

The cell lines were stably transduced with a lentivirus vector expressing the enhanced Green Fluorescent Protein (eGFP) (3). Knock-down of *SET* was conducted *in vitro* using the lentiviral viruses purchased from Sigma (supplementary table 11). The viruses are based on the plasmid vector pLKO.p1 co-expressing a puromycin resistance cassette to enable transduction selection of mammalian cells and ensure the establishment of stable clones. As a transduction negative control, we used non targeting shRNA control (shScramble), designed to target no known gene sequence in the cells being used. The pLKO, 1-puro CMV-tag RFP, with no shRNA insert and expressing the Red Fluorescent Protein, was used as a transduction positive control. For the transduction, the cells were split the day before and maintained at the concentration of 2x10^5^ cells/mL. The following day, the cells were harvested and counted. For each transduction, 20,000 cell/well were plated in a 96-wells plate U bottom, in a volume of 100μL of transduction mix containing fresh medium, lentiviral particles (1-5 MOI) and polybrene (5 µg/mL). After gently resuspending the transduction mix, the cells were spun down at 800 g for 15 minutes at 32^o^C and then incubated at 37^o^C in humidified 5% CO_2_ atmosphere. After sixteen hours, the plate was removed from the incubator and 100µL of pre-warmed complete culture medium were added to the 100µL of the cell suspension for a total volume of 200µL. Then, incubated again at 37^o^C in humidified 5% CO_2_ atmosphere for additional 24 hours. Forty-eight hours after the transduction, the cells were transferred to Eppendorf tubes previously dispensed with 800μL of pre-warmed semi-solid methylcellulose- based medium (Stem Cell Technology, M3231), supplemented with 100 IU/mL penicillin and 100 mg/mL streptomycin and an appropriate amount of puromycin for selection. The cells were seeded into a 24-well plate and incubated at 37^o^C humidified 5% CO_2_ incubator for 7- 14 days until colonies of cells appeared. During this period, the plate was scanned under the microscope to evaluate the relative distribution of the colonies over the time. After 7-10 days, the colonies were scored and dispersed in PBS/0.5%FBS. 2,000-5,000 cells were subsequently re-plated in 800μL of methylcellulose supplemented with puromycin and incubated up to 10-14 days. This procedure was repeated for three rounds to determine the impact of SET KD on colony forming unit (CFU). The method employed was adjusted according to the retroviral/lentiviral transduction and transformation assay protocol (RTTA/LTTA) (4). The transduction efficiency was determined using red fluorescence emission from RFP+ cells. After 48 hours from the transduction, cells were gently resuspended and 10µL of untransduced and CMV-tag RFP cells (RFP+) were added on a glass side and analysed under EVOS Fluorescence Microscope to track green and red fluorescence at 10X magnification.

**Immunoblotting**

Samples for immunoblot analysis were lysed by sonication in RIPA buffer supplemented with protease inhibitors (10μg/mL of aprotinin, leupeptin, antipain, soybean inhibitor and 1mM phenylmehylsulfony fluoride PMSF) and phosphatase inhibitors (50mM sodium fluoride, 1mM sodium orthovanadate). The homogenate was centrifuged at 16,000 x g for 15 minutes at 4^o^C to remove the insoluble material. The extract was collected and assayed in triplicate for protein quantification using Bradford assay kit (Sigma), mixing one part (5µL) of the protein sample with 50 parts (250µL) of the Bradford reagent. The reaction was incubated on a shaker at room temperature for 10 minutes and the absorbance was detected using Multiskan EX microplate reader (Thermo fisher) at 595 nm. The protein concentration was then determined by interpolation on a nine serial dilutions BSA standard curve generated in water within the concentration range of 2mg/mL to 0.1mg/mL. Lysates were heated to 95^o^C in SDS sample buffer supplemented with 100 mM dTT for 5 minutes, separated by SDS-PAGE and transferred to PVDF membranes 0.2µm pore size (Amersham™). Membranes were blocked in 5% non-fat dry milk in TBS + 0.1% Tween-20 probed with the indicated antibodies (supplementary table 12 and 13) and reactive bands were visualized using ECL Prime (Pierce), according to the manufacturer’s instructions. Band densities detection was obtained using Odyssey ® Fc Imaging System, LI-COR Biosciences.

### **Nuclear Cytoplasmic fractionation**

Lysates were prepared from a pellet of 5x10^6^cells, using a specific volume of Nuclear and Cytoplasmic extraction reagents from NE-PER™ (Thermo Scientific), according to the manufacture’s protocol. Briefly, for cytoplasmic extraction the sample was initially re-suspended with cytoplasmic extraction reagent (CER I), supplemented with protease and phosphatase inhibitors, vortexed and incubated in ice for 10 minutes. In order to collect the cytoplasmic fraction, the tube was centrifuged at 16,000 x g for 15 minutes and the supernatant was transferred to a pre-chilled tube. The insoluble pellet containing the nuclei, was further incubated on ice for 40 minutes with nuclear lysis buffer (NER). The nuclear lysate was centrifuged at maximum speed in a microcentrifuge for 10 minutes, transferred to a pre-chilled tube and stored at -80^o^C. As final stage, the isolated proteins were quantified using Bradford assay kit and analysed by immunoblot for SET, GAPDH and Laminin B1 detection as described.

### **Co-Immunoprecipitation (Co-IP) assay**

For SET-PP2A co immunoprecipitation, cells were lysed in NP40 buffer (100 mM Tris, 0.2% NP40, 150 mM NaCl, pH 7.4) (5), with protease inhibitors (10 μg/mL aprotinin, 10 ug/mL soybean inhibitor, 1mM PMSF). The lysate was incubated on ice for 15 minutes and centrifugated at 16,000 x g for 15 minutes at 4^o^C to remove the insoluble material. After collecting the clear supernatant, the protein concentration was quantified using Bradford assay kit as described above. Protein concentration of the lysate was adjusted to 400 μg of total protein in 500 μL of lysis buffer and aliquoted to two different tubes for co-immunoprecipitation assay and negative control of co-immunoprecipitation assay. From each tube, 5 μL of lysate were aliquoted as input to confirm target expression within the total protein lysate. The samples were stored at -20 ^o^C with SDS sample buffer 5X. The rest of the lysate was incubated with the antibody anti-PP2Ac (1:100) at 4^o^C in constant agitation on a wheel, overnight. The negative control was incubated with no antibody . The following day, magnetic Dynabeads (Millipore) were carefully re-suspended on a roller for 5 minutes and quickly washed three times with lysis buffer. The washes were performed on a magnetic rack which allowed to separate the beads from the lysis buffer and discard it. Next, 25 μL of them were added to lysate/peptide mixture and negative control tubes for 3 hours at 4^o^C in constant agitation. After the incubation, the tubes were placed on a magnetic separation rack and, before discarding the supertanat, 20 μL of mixture were aliquoted to a clean tube. This sample represented the flowthrough control wich confirmed whether the antigen or binding partners were bound to anti-PP2Ac antibody. The lysate mixtures were washed three times with 200μL of chilled phosphatase buffered saline PBS (0.137 M NaCl, 0.0027 M KCl, 0.01 M Na_2_HPO_4_, 0.0018 M KH_2_PO_4,_ pH 7.4) gently pipetting. The tubes were placed on a magnetic separation rack to separate the lysate/peptide/beads mixture from the washing buffer. From the first two washes, 2 μL of supernatant were collected as wash buffer controls and supplemented with SDS sample buffer 5X for Western blot analysis. The rest of the wash buffer was discarded. The co-immunoprecipitated complex was eluted from the magnetic beads adding 30 μL of SDS sample buffer 5X at 96^o^C for 5 minutes. After a brief centrifugation, the tubes were placed on the magnetic separation rack and the supernatant, representing the co-immunoprecipitated complex, was collected. Following that step, 30 μL of SDS sample buffer 5X were added to the beads and then heated at 96^o^C for 5 minutes. This control was representative of elution efficiency. The negative Co-IP control was processed in the same way. Input, flowthrough, wash buffer controls and beads were heated under reducing conditions at 96^o^C for 5 minutes and analyzed by western blot with the co-immunoprecipiated sample. Western blot analysis was conducted as described above. The membranes were incubated with primary antibody anti-SET overnight at 4^o^C in order to asses whether SET was binding PP2A forming a protein- protein interaction complex. Immunoprecipitation assay was also used to isolate phosphorylated forms of SET. SET was immunoprecipitated using the antibody anti-SET (1:100) according to immunoprecipitation protocol described above. After that, serine phosphorylation of SET was detected by western blot analysis using anti- Phosphor-SER as primary antibody.

For SET-MLL co-IP cells were lysed by incubation for 30 minutes at 4^o^C in lysis buffer [50 mM Tris/HCl pH 8, 150 mM NaCl, 0.5% NP-40, 2 mM EDTA pH 8, 1 mM Na_3_V0_4_, 10 mM NaF, 0.1% (v/v) protease inhibitor cocktail (PIC) (Sigma Aldrich)]. Cell lysates were then centrifuged at 16,000 g at 4^o^C for 5 minutes and the supernatants consisting of whole-cell protein extracts (WCE) were used for Co-IP experiments. WCEs were incubated overnight at 4^o^C, under gentle shaking, with a mouse monoclonal specific antibody (Ab) anti-MLL (6, 7) or anti-SET or with total mouse IgGs (as a control), using 2 μg per 5 mg of total proteins. The protein mixtures were then incubated for 60 minutes at 4^o^C, under gentle shaking, with protein A/G PLUS-Agarose beads (Santa Cruz Biotechnology) using 30 μL per 1 μg antibody. After several washes with lysis buffer, the immunocomplexes-bound beads were precipitated by centrifugation at 10,000 g at 4^o^C, were resuspended in 30 μL of Laemmli buffer (Bio-Rad Laboratories) to constitute the IP samples. Either 40 μg of WCE or 15 μl of IP sample were loaded onto SDS/PAGE. After electrophoretic separation, the proteins were transferred by electroblotting onto nitrocellulose membranes and analyzed using anti-MLL or anti-SET primary Abs and horseradish peroxidase-conjugated secondary Abs. The signals were visualized using an ECL Plus detection system (GE Healthcare).

**RNA extraction**

The total RNA from THP1, MV411 and SEM shscramble and shSET was extracted using the RNA mini kit Zymoresearch. For all the other samples the total RNA was extracted using Isolate II RNA mini kit (Bioline). The samples were lysed with guanidinium thiocyanate and processed through a spin column of silica membrane for RNA binding. Genomic DNA contamination, cellular components and impurities were removed through DNase I digestion and washing the columns with the supplied buffers. Final RNA was eluted 40 μL of RNase- free water. Quantification, integrity and purity of RNA was measured on NanoVue™ Plus Spectrophotometer. Integrity and purity of the RNA was assessed based on 260/280 nm and 260/230 absorbance ratio.

### **Reverse transcription**

Two µg of RNA were reverse transcribed using SensiFAST™ cDNA Synthesis Kit (Bioline) in 20uL volume. The master mix reaction was prepared on ice as described below and the reaction was set up as follows: primer annealing 25^o^C for 10 minutes, reverse transcription 42^o^C for 15 minutes, inactivation 85^o^C for 5 minutes and final hold at 4^o^C. The cDNA generated was store at -20^o^C for real time PCR analysis.

### **Real time PCR**

Quantitative real time PCR (RT-qPCR) was performed using specific primers from SIGMA (supplementary table 14)*.* Quantitative real-time PCR was carried out using SensiFAST™ SYBR® No-ROX Kit (Bioline), using the primers at a final concentration of 0.8 µM. The final concentration of cDNA used for each experiment, depended on the expression of each individual target but essentially 20 ng of cDNA were used for most of them. The reaction was conducted in The StepOnePlus™ Real Time System instrument (Applied Biosystem) with initial holding stage at 95^o^C for 2 minutes, followed by 40 amplification cycles at 95^o^C (denaturation) for 30 seconds, 60^o^C (annealing) for 10 seconds and at 72^o^C (extension) for 20 seconds, with a single fluorescence measurement, and a final dissociation step (95^o^C for 30 seconds, 65^o^C for 30 seconds, 95^o^C for 30 seconds) and cooling holding at 40^o^C .To optimize the RT-PCR conditions, the efficiency of the primers was determined using a standard curve, generated from 1:10 dilution series of sample nucleic acid, known as calibrator, and processed through real time PCR. GAPDH was used as housekeeping control for gene normalisation and loaded along with each target in one plate. Relative gene expression was quantified using the Pfaffl formula to account for differences in primers efficiencies (8).

**Chromatin immunoprecipitation (ChIP) assay**

ChIP assay was performed as previously reported (6, 7). Briefly, cells (30 × 10^6^) were fixed by incubation for 10 minutes at room temperature in culture medium containing 1% formaldehyde for 10 minutes at room temperature, and the reaction was then stopped by glycine quenching (125 mM final concentration). Fixed cells were washed twice in PBS, collected by centrifugation, and then resuspended and incubated in cell lysis buffer (5mM PIPES pH 8.0, 85 mM KCl, 0.5% NP-40, 0.1% PIC) for 10 minutes at 4^o^C. The suspension was centrifuged for 5 minutes at 4^o^C at 1,000 g in order to precipitate the nuclei. The nuclei were resuspended and lysed by incubation in nuclei lysis buffer (50 mM Tris-HCl pH 8.1, 10 mM EDTA, 0.8% SDS, 0.1% PIC) for 10 minutes at 4^o^C. To fragment the chromatin, the nuclei lysate was sonicated at 4^o^C (6 cycles of 30 seconds of sonication interspersed by pauses of 30 seconds) using a Microson XL ultrasonic cell disruptor (Misonix, Farmingdale, NY) and then centrifuged for 10 minutes at 4^o^C at 20,000 g. The supernatant containing protein-bound DNA fragments ranging from 100 to 600 bp was diluted with 3 volumes of dilution buffer (1% Triton, 0.5 mM EGTA, 10 mM Tris-HCl pH 8.1, 140 mM NaCl, 0.1% PIC). A proper amount of the supernatant was added to 1 volume of ChIP buffer (0.1% SDS, 1% Triton, 1 mM EDTA, 0.5 mM EGTA, 10 mM Tris-HCl pH 8.1, 140 mM NaCl, 1 % Na-deoxycholate) and centrifuged for 10 minutes at 4^o^C at 20,000 g. The supernatant was incubated overnight at 4°C with specific antibodies (anti-MLL, anti-SET) or mouse IgG (as a control), under gentle shaking, then mixed with protein A/G PLUS-Agarose beads (Santa Cruz Biotechnology) and incubated again for 120 minutes at 4^o^C, under gentle shaking. The beads were washed several times with ChIP buffer, LiCl buffer (0.25 M LiCl, 0.5% NP-40, 0.5% Na-deoxycholate, 1 mM EDTA, 10 mM Tris-HCl pH 8.1) and TE buffer (1 mM EDTA, 10 mM Tris-HCl pH 8.1), resuspended in TE buffer and incubated overnight at 65^o^C with 10 ng/µL RNAse (Thermo Fisher). 0.5 µg/ µL proteinase K and 0.5% SDS were added to the sample that was incubated 4 hours at 50^o^C and then centrifuged for 2 minutes at 4^o^C at 15,000 g, in order to precipitate the resin. DNA was harvested from the recovered supernatant by phenol/chloroform/isoamyl alcohol extraction and ethanol precipitation. qPCR was performed with 1 μl DNA using custom-made primer sets. In particular, the *HOXA9* promoter (HOXA9-pr) and the *ACTB* promoter (ACTB-pr) were amplified with a previously reported primer pair (6, 7), while the three tested *HOXA10* promoter elements, namely HOXA10-prE1, HOXA10-prE2 and HOXA10-prE3) were amplified with primer pairs reported in Supplementary Table 15. The primers for HOXA10-prE1 and for HOXA10-prE3 were reported by (9, 10), while those for HOXA10-prE2 were designed to amplify a sequence that was reported as MLL-binding element by (11).

## **Cell proliferation analysis**

The cell lines were stably transduced with a lentivirus vector expressing the enhanced Green Fluorescent Protein (eGFP) and positive clones were sorted by FACS, as described (3). Cells were plated at cell density ranging from 5x10^4^ to 1x10^5^ cells/mL in 6 well plates in complete medium and incubated at 37^o^C humidified 5% CO_2_ atmosphere_._  Cell proliferation was monitored on the day when the cells were seeded and then every two days up until 6 days post seed, by measuring GFP fluorescence at a Biotek fluorescent microplate reader at 495 nm and by colorimetric method, performing the Cell Titer Aqueous Cell Proliferation (MTS) assay and measuring the optical density (OD) at 490 nm. The data of the proliferation assay based on GFP monitoring, were reported as RFU (Fluorescence Units) versus days of incubation. The data of the proliferation assay based on MTS assay, were reported as OD (Optical density) versus days of incubation. The correlation between the results of the two proliferation assays, the one based on GFP monitoring and the one based on the MTS assay, was calculated by Pearson’s correlation on Graph Pad Prism. The Pearson’s R square are reported in the panels of supplementary Fig. 3 are above 0.8.

**Calculation of FTY720 IC50**

FTY720 was purchased from Selleckchem (S5002) and resuspended in DMSO. Alive cells were discriminated by trypan blue exclusion. The IC50 was calculated in Graph Pad Prism.

**Cell death analysis**

The effect of 5 μM FTY720 treatment on cell death was analysed by FACS. The day before the treatment, the cells were maintained in culture at a concentration of 2x10^5^ cells/mL. The following day, the cells were harvested, centrifuged at 500 g for 5 minutes and counted. For each condition the cell suspension was adjusted to 20,000 cells in 100μL of complete medium and plated in triplicate in three 96 well-plates flat bottom for 72 hrs for cell death analysis, as reported (3). Briefly, the samples were processed using BD Accuri™ C6 Flow cytometer and analysed for expression of GFP, measured in FL1-A channel (533/30). The cell population was identified and gated based on the forward and side scatter signals profile (FSC/SSC). The combination of these two parameters allows for the discrimination of the cells by size and internal complexity in single-cell analysis. Among this population, we performed doublets discrimination by plotting area (-A) against the height (-H) for side scatters (SSC-A versus SSC-H). Doublets present double the area and width values of single cells whilst the height is roughly the same. Disproportions between height and area are used to identify doublets. This accurate discrimination ensures the exclusion of false GFP emission. For each cell population, we recorded a minimum of 10,000 events in fast flow rate. Results were given in percentage of GFP positive cells.

For each cell cycle assay 1x10^5^ cells were collected, washed in PBS and fixed in 70% cold ethanol. After re-hydration with PBS and centrifugation at 500g for five minutes, the cells were incubated with a solution of PBS containing 1% FCS, 40ug/ml RNAse and 500ug/ml propidium iodide solution (Sigma-Aldrich) in the dark for 30 minutes at 37^o^C. DNA peaks were analyzed with BD AccuriC6 by collecting the fluorescence on FL2. For each cell population, we recorded a minimum of 10,000 events in slow flow rate. The instrument was calibrated according to the standard BD Accuri C6 protocol using BD Accuri™ Spherotech 8-Peak Validation beads (FL1, FL2 and FL3 channels) and 6-Peak Validation beads (FL4 channel).

**May-Grunwald-Giemsa staining**

May- Grunwald Giemsa staining was performed as described (12). 1x10^5^ cells were cytospun for 5 minutes at 300 g onto glass slides. Slides were then stained with May-Grunwald solution (Sigma-Aldrich) for 3 minutes at room temperature. After washing in water, they were incubated for 20 minutes in Giemsa solution (Sigma-Aldrich) (1:20 in water). Slides were washed again in water before being mounted with Mowiol.

**Phospho-proteomic experiments**

Phospho-proteomic experiments were performed using mass spectrometry with some technical modifications as reported (13, 14). In brief, frozen cell pellets were lysed in 8M urea buffer and supplemented with phosphatase inhibitors (10 mM Na_3_VO_4_, 100 mM β-glycerol phosphate and 25 mM Na_2_H_2_P_2_O_7_ (Sigma)). Proteins were digested into peptides using trypsin as previously described (15, 16). Phosphopeptides were desalted and enriched using the AssayMAP Bravo (Agilent Technologies) platform. For desalting, protocol peptide clean-up v3.0 was used. Reverse phase S cartridges (Agilent, 5 μL bed volume) were primed with 250 μL 99.9% acetonitrile (ACN) with 0.1%TFA and equilibrated with 250 μL 0.1% TFA at a flow rate of 10 μL/min. The samples were loaded at 20 μL/min, followed by an internal cartridge wash with 0.1% TFA at a flow rate of 10 μL/min. Peptides were then eluted with 105 μL of 1M glycolic acid with 50% ACN, 5% TFA and this is the same buffer for subsequent phosphopeptide enrichment. Following the Phospho Enrichment v 2.1 protocol, phosphopeptides were enriched using 5ul Assay MAP TiO2 cartridges on the Assay MAP Bravo platform. The cartridges were primed with 100ul of 5% ammonia solution with 15% ACN at a flow rate of 300 μL/min and equilibrated with 50 μL loading buffer (1M glycolic acid with 80% ACN, 5% TFA) at 10 μL/min. Samples eluted from the desalting were loaded onto the cartridge at 3 μL/min. The cartridges were washed with 50 μL loading buffer and the phosphorylated peptides were eluted with 25 μL 5% ammonia solution with 15% ACN directly into 25 μL 10% formic acid. Phosphopeptides were lyophilized in a vacuum concentrator and stored at -80^o^C.

Dried phosphopeptides were dissolved in 0.1% TFA and analysed by nanoflow ultimate 3000 RSL nano instrument was coupled on-line to a Q Exactive plus mass spectrometer (Thermo Fisher Scientific). Gradient elution was from 3% to 28% solvent B in 90 min at a flow rate 250 nL/min with solvent A being used to balance the mobile phase (buffer A was 0.1% formic acid in water and B was 0.1% formic acid in acetonitrile). The spray voltage was 1.95 kV and the capillary temperature was set to 255^o^C. The Q-Exactive plus was operated in data dependent mode with one survey MS scan followed by 15 MS/MS scans. The full scans were acquired in the mass analyser at 375- 1500m/z with the resolution of 70 000, and the MS/MS scans were obtained with a resolution of 17 500.

MS raw files were converted into Mascot Generic Format using Mascot Distiller (version 2.6.1) and searched against the SwissProt database (SwissProt_2020_04) restricted to human entries using the Mascot search daemon (version 2.6.1). Allowed mass windows were 10 ppm and 25 mmu for parent and fragment mass to charge values, respectively. Variable modifications included in searches were oxidation of methionine, pyro-glu (N-term) and phosphorylation of serine, threonine and tyrosine. Phosphopeptide quantification was performed using in-house software Pescal as described before (15). The resulting quantitative data was parsed into excel files for further normalisation and statistical analysis.

Samples were analysed in technical duplicated and the mean average intensity of phophopeptides calculated. Data was further analysed and visualised using phosphomatics (17). Phosphopeptides were normalised by overall sample intensity and Log2 transformed, value filtering and data imputation were not performed. Substrate kinase interactions were inferred using PhosphoSitePlus (18) and SIGNOR 2.0 (19).Kinase-Substrate Enrichment Analysis (KSEA) was performed using KSEA App (20). For a given upstream kinase, the m threshold was set to 5, this is the minimum number of substrates assigned to a given kinase for it to be included in KSEA analysis. The NetworKIN PhosphoSitePlus threshold was set to 5 and the p-value cut off was <0.1. Gene ontology enrichment analysis was performed using Database for Annotation, Visualization and Integrated Discovery (DAVID) Gene Ontology (21, 22) and visualised using Cytoscape v3.9.1 (23). Principal component analysis was performed in Phosphomatics and sample correlation analysis was performed using Spearman rank. Substrate clustered heatmaps were generated using either Phosphomatics or ClustVis (24). To generate the heatmaps average linkage and Euclidean distance hierarchical clustering to create groups of phosphorylation sites that share similar patterns of abundance changes. Log2 fold-change of >1 and p-value <0.1 filters were applied to exclude phosphosites that were unchanged in abundance and phosphosite abundances were z-transformed by row. Volcano plots were produced using Phosphomatics to identify phosphosites that are highly significantly changed by treatment with FY720. For each phosphosite, the log2(fold-change) between two groups was plotted on the x-axis and the -Log10(p-value) for the observation on the y-axis. Points, corresponding to individual phosphosites, appearing in the upper left and upper right quadrants of the plot possess a fold change >2 and p-value <0.01. Protein interaction networks were generated using a custom database manually curated based on the PICKLE2.0 human protein-protein interaction meta-database (25). These data were visualised using Cytoscape v3.9.1.

**RNA-seq Bioinformatic Analysis**

The RNA seq in FTY720 vs vehicle-treated THP1 was performed by Novogene. Downstream analysis was performed using a combination of programs including STAR, HTseq, Cufflink and Novogene wrapped scripts. Alignments were parsed using Tophat program and differential expressions were determined through DESeq2/edgeR. GO and KEGG enrichment were implemented by the ClusterProfiler. Gene fusion and difference of alternative splicing event were detected by Star-fusion and rMATS software. Reference genome and gene model annotation files were downloaded from genome website browser (NCBI/UCSC/Ensembl) directly. Indexes of the reference genome was built using STAR and paired-end clean reads were aligned to the reference genome using STAR (v2.5) (26 ). STAR used the method of Maximal Mappable Prefix(MMP) which can generate a precise mapping result for junction reads. HTSeq v0.6.1 was used to count the read numbers mapped of each gene. And then FPKM of each gene was calculated based on the length of the gene and reads count mapped to this gene. FPKM, Reads Per Kilobase of exon model per Million mapped reads, considers the effect of sequencing depth and gene length for the reads count at the same time, and is currently the most commonly used method for estimating gene expression levels (27). Differential expression analysis between two conditions/groups (three biological replicates per condition) was performed using the DESeq2 R package (2_1.6.3) (28, 29). DESeq2 provide statistical routines for determining differential expression in digital gene expression data using a model based on the negative binomial distribution. The resulting P-values were adjusted using the Benjamini and Hochberg’s approach for controlling the False Discovery Rate(FDR). Genes with an adjusted P-value <0.05 found by DESeq2 were assigned as differentially expressed. Prior to differential gene expression analysis, for each sequenced library, the read counts were adjusted by edgeR program package through one scaling normalized factor (30). Differential expression analysis of two conditions was performed using the edgeR R package (3.16.5). The P values were adjusted using the Benjamini & Hochberg method. Corrected P-value of 0.05 and absolute foldchange of 1 were set as the threshold for significantly differential expression. The Venn diagrams were prepared using the function venn Diagram in R based on the gene list for different group. Gene Ontology (GO) enrichment analysis of differentially expressed genes was implemented by the cluster Profiler R package, in which gene length bias was corrected. GO terms with corrected P value less than 0.05 were considered significantly enriched by differential expressed genes. KEGG is a database resource for understanding high-level functions and utilities of the biological system, such as the cell, the organism and the ecosystem, from molecular level information, especially large-scale molecular datasets generated by genome sequencing and other high-through put experimental technologies (<http://www.genome.jp/kegg/>) (31). We used cluster Profiler R package to test the statistical enrichment of differential expression genes in KEGG pathways (32). PPI analysis of differentially expressed genes was based on the STRING database, which contained known and predicted Protein-Protein Interactions. For the species existing in the database (like human and mouse), we constructed the networks by extracting the target gene lists from the database.

## **Statistical analysis**

Densitometry of immunoblotting images was performed using Image Studio software and normalised by Microsoft Excel, relatively to the loading control’s expression. Statistical significance was determined using GraphPad Prism 7.

**Supplementary table 8 Characteristics of cell lines**

| **Cell type** | **Cell line** | **Fusion gene** | **Translocation** | **Origin** | **Source** |
| --- | --- | --- | --- | --- | --- |
| **KMT2A-R** | THP1 | MLL-AF9 | t(9;11) | 1-years old boy with AML | Gift from Dr Yolanda Calle |
|  | MV4;11 | MLL-AF4 | t(4;11) | 10-years old boy with AML | Gift from Prof. Eric So |
|  | ML2 | MLL- AF6 | t(6;11) | 26-years old woman with AML | DSMZ |
|  | NOMO1 | MLL-AF9 | t(9;11) | 31-years old woman with AML | DSMZ |
| **KMT2A-R** | SEM1 | MLL-AF4 | t(4;11) | 5-years old girl with ALL | Gift from Prof. Eric So |
|  | Hb11;19 | MLL-ENL | t(11;19) | (Age not specified) ALL | Gift from Prof. Eric So |
|  | RS4;11 | MLL-AF4 | t(4;11) | 36-years female with ALL | DMSZ |
|  | KOPN8 | MLL-AF4 | t(11;19) | 3- months- old girls with ALL | DSMZ |
| **KMT2A-wt** | K562 | BCR-ABL | t(9;22) | 53-years female with CML | Gift from  Dr Yolanda Calle |
| **KMT2A-wt** | Kasumi1 | AML1-ETO | t(8;21) | 7-years female with AML | Gift from Dr Yolanda Calle |
| **KMT2A-wt** | REH | TEL-AML1 | t(12;21) | 15-years female with ALL | Gift from Dr Owen Williams |
| **KMT2A-wt** | U937 | MLLT10-PICALM | t(10;11) | 37-years male with HL | Gift from Dr David Guiliano |

**Supplementary table 9 Characteristics of the primary samples**

| **Sample** | **Disease** | **Sample Type** | **Cytogenetics** |
| --- | --- | --- | --- |
| 1 | AML | Leucopheresis | INS (10;11)(P12,Q23Q21) MLL Abnormality |
| 2 | AML | Peripheral blood | t(11;17) MLL (11q23) |
| 3 | AML | Peripheral blood | t(9;11) |
| 4 | AML | Bone marrow | t(9;11) |
| 5 | AML | Peripheral blood | t(9;11) |
| 6 | AML | Bone marrow | t(6;11) MLL-MLL t4 rearrangement |
| 7 | AML | Peripheral blood | t(6;11) (Q27;232323) |
| 8 | ALL | Peripheral blood | t(4;11) |
| 9 | ALL | Peripheral blood | t(4;11) |

| **Supplementary table 10 Summary of of human bone marrow and peripheral blood mononuclear cells** |
| --- |

| **Sample** | **Cell type** | **Origin** | **Age** | **Sex** | **Ethnicity** | **Weight**  **(kg)** | **Height**  **(cm)** | **Smoker** |
| --- | --- | --- | --- | --- | --- | --- | --- | --- |
| 1 | MNCs | Bone Marrow | 26 | Female | Caucasian | 72 | 177 | no |
| 2 | MNCs | Bone Marrow | 52 | Female | Caucasian | 69 | 165 | no |
| 3 | MNCs | Bone Marrow | 36 | Female | Caucasian | 59 | 163 | no |
| 4 | MNCs | Bone Marrow | 25 | Female | Hispanica | 57 | 151 | no |
| 5 | MNCs | Bone Marrow | 20 | Male | Caucasian | 66 | 168 | no |
| 6 | MNCs | Bone Marrow | 37 | Male | Caucasian | 77 | 170 | no |
| 7 | MNCs | Bone Marrow | 37 | Male | Caucasian | 59 | 163 | no |
| **Sample** | **Cell type** | **Origin** | **Age** | **Sex** | **Ethnicity** | **Weight**  **(kg)** | **Height**  **(cm)** | **Smoker** |
| 1 | MNC | Peripheral Blood | 26 | Female | Caucasian | 72 | 177 | no |
| 2 | MNC | Peripheral Blood | 52 | Female | Caucasian | 69 | 165 | no |
| 3 | MNC | Peripheral Blood | 36 | Female | Caucasian | 59 | 163 | no |
| 4 | MNC | Peripheral Blood | 25 | Female | Hispanica | 57 | 151 | no |

**Supplementary table 11. Summary of shRNA**

| **Lentiviral**  **transduction particles** | **INSERT SEQUENCE/DESCRIPTION** | **Insert Sequence (5’-3’)** |
| --- | --- | --- |
| **SHC002V**  MISSION non-mammalian pLKO, 1-puro shRNA Control Transduction Particles | No human or mouse shRNA | CAACAAGATGAAGAGCACCAA |
| **SHC012V**  pLKO, 1-puro CMV-tag RFP control transduction particles | No human or mouse shRNA  Contains TagRFP gene under the control of the CMV promoter. | No hairpin |
| **SHCLNV**  **(TRCN000063716)** | shRNA against *SET*  NM_ID 003011 | GCGATTGAACACATTGATGAA |
| **SHCLNV**  **(TRCN0000380015)** | shRNA against *PPP2CA*  NM_ID 002715 | ACCGGAATGTAGTAACGATTT |

**Supplementary table 12. List of Primary Antibodies used for the study**

| **Primary antibody** | **Molecular weight** | **Dilution** | **Species** | **Company/Catalogue number** |
| --- | --- | --- | --- | --- |
| SET | 39kDa | 1:500  in 5% non- fat dry milk | Mouse | Santacruz / 133138 |
| c-Myc | 57-70kDa | 1:500  in 5% BSA | Rabbit | Cell signalling / 9402S |
| Phospho-c-Myc Thr58 | 60 KDa | 1:500  in 5% BSA | Rabbit | Cell signalling/46650 |
| Phospho c-Myc  Ser62 | 62kDa | 1:500  in 5% BSA | Rabbit | Cell signalling / 13748 |
| PLK1 | 58kDa | 1:500  in 5% milk | Mouse | Santacruz/17783 |
| Phospho-PLK1 Thr210 | 58kDa | 1:500  in 5% BSA | Rabbit | Cell signalling/5472 |
| GAPDH | 37kDa | 1:1000  in 5% non- fat dry milk | Rabbit | Cell signalling / 2118 |
| Laminin B1 | 67kDa | 1:1000  in 5% non-fat dry milk | Mouse | Santa Cruz / 374015 |
| Phospho-Ser | * | 1:500  in 5% BSA | Mouse | Santa Cruz /81514 |
| AKT (pan) | 60KDa | 1:2000  in 5% non-fat dry milk | rabbit | Cell Signalling Technology/4691 |
| Phosphor AKT1/2  Ser473 | 60KDa | 1:1000  in 5% BSA | rabbit | Cell Signalling Technology/4060 |
| Phospho AKT1/2  Thr308 | 60KDa | 1:1000  in 5% BSA | rabbit | Cell Signalling/13038 |
| GSK3Beta | 46KDa | 1:1000  in 5% non-fat dry milk | rabbit | Cell Signalling Technology/9832 |
| GSK3Beta  Ser9 | 46KDa | 1:1000  in 5% BSA | rabbit | Cell Signalling Technology/5558 |
| ERK1/2 | 42-44 KDa | 1:1000  in 5% non-fat dry milk | mouse | Santacruz/  sc514302 |
| Phosphor-ERK1/2 Thr202/TYR204 | 42-44KDa | 1:1000  in 5% BSA | rabbit | Cell Signalling Technology/9101 |
| PP2A | 36 KDa | 1:1000  in 5% non-fat dry milk | mouse | Sigma/ 05-421 |
| MLL | 180KDa | 1:1000  in 5% non-fat dry milk | mouse | Sigma 05-765 |
| IgG control |  | 1:1000  in 5% non-fat dry milk | mouse | Santacruz/ sc-2025 |

**Depending on the targets which presents the phosphorylation in question*

**Supplementary table 13. List of Secondary Antibodies used for the study**

| **Secondary Antibody** | **Dilution** | **Company** |
| --- | --- | --- |
| (HRP)-conjugated anti-mouse | 1:10000 in 5% non-fat dry milk | Jackson Immunoresearch |
| (HRP)-conjugated anti-rabbit | 1:10000 in 5% non-fat dry milk | Jackson Immunoresearch |
| IRDye secondary antibody anti-mouse 800 CW | 1:5000 in 5% non-fat dry milk | LI-COR |
| IRDye secondary antibody anti-rabbit 680 RD | 1:5000 in 5% non-fat dry milk | LI-COR |

**Supplementary table 14. List of primers used for the qPCR experiments**

| **GENE OF INTEREST** | **Primer direction** | **SEQUENCE** | **ACCESSION NUMBER** |
| --- | --- | --- | --- |
| GAPDH | Forward | 5’- GAG GTC AAT GAA GGG -3’ | NM_002046.7 |
|  | Reverse | 5’- AGG TGA AGG TCG GAG -3’ |  |
| SET | Forward | 5’ – TGG TTG GCG GAG TTT -3’ | NM_003011.4 |
|  | Reverse | 5’- AGC AAG CGA TTG -3’ |  |
| MYC | Forward | 5’-CTAACAGAAATGTCCTGAGC-3’ | NM_002467 |
|  | Reverse | 5’TCCAATTTGAGGCAGTTTAC-3’ |  |
| HOXA10 | Forward | 5’-GAGAAGGGAGACATTGTTTG-3’ | NM_018951 |
|  | Reverse | 5’-TTTCTGCATCTACAGGTTTG-3’ |  |
| HOXA9 | Forward | \| 5’-gccggccttatggcattaa-3’ \| \| --- \| \|  \| | NM_152739 |
|  | Reverse | 5’-cagggacaaagtgtgagtgtcaa-3’ |  |

**Supplementary table 15. List of primers used for the ChiP experiments**

| ***HOXA10* promoter elements** | **Direction** | **Sequence** |
| --- | --- | --- |
| HOXA10-prE1 | Forward | 5’-GGATCAACGGACTAGGGGAGA-3’ |
|  | Reverse | 5’-CCTCAAAAGTGGCGAACCTG-3’ |
| HOXA10-prE2 | Forward | 5’-AATCGCGGGCTCTGAGGGC-3’ |
|  | Reverse | 5’-CCTTTCTGGCTGACATTTCTTG-3’ |
| HOXA10-prE3 | Forward | 5’-CGCAACCACCCCAGCCAG-3’ |
|  | Reverse | 5’-TTGTCCGCCGAGTCGTAGAGG-3’ |

**Additional references**

1. Walf-Vorderwulbecke V, Pearce K, Brooks T, Hubank M, van den Heuvel-Eibrink MM, Zwaan CM, et al. Targeting acute myeloid leukemia by drug-induced c-MYB degradation. Leukemia. 2018;32(4):882-9.

2. Clesham K, Walf-Vorderwulbecke V, Gasparoli L, Virely C, Cantilena S, Tsakaneli A, et al. Identification of a c-MYB-directed therapeutic for acute myeloid leukemia. Leukemia. 2022;36(6):1541-9.

3. Arroyo-Berdugo Y, Sendino M, Greaves D, Nojszewska N, Idilli O, So CW, et al. High Throughput Fluorescence-Based In Vitro Experimental Platform for the Identification of Effective Therapies to Overcome Tumour Microenvironment-Mediated Drug Resistance in AML. Cancers (Basel). 2023;15(7).

4. Zeisig BB, So CW. Retroviral/Lentiviral transduction and transformation assay. Methods Mol Biol. 2009;538:207-29.

5. Switzer CH, Cheng RY, Vitek TM, Christensen DJ, Wink DA, Vitek MP. Targeting SET/I(2)PP2A oncoprotein functions as a multi-pathway strategy for cancer therapy. Oncogene. 2011;30(22):2504-13.

6. Fioretti T, Cevenini A, Zanobio M, Raia M, Sarnataro D, Salvatore F, et al. Crosstalk between 14-3-3theta and AF4 enhances MLL-AF4 activity and promotes leukemia cell proliferation. Cell Oncol (Dordr). 2019;42(6):829-45.

7. Fioretti T, Cevenini A, Zanobio M, Raia M, Sarnataro D, Cattaneo F, et al. Nuclear FGFR2 Interacts with the MLL-AF4 Oncogenic Chimera and Positively Regulates HOXA9 Gene Expression in t(4;11) Leukemia Cells. Int J Mol Sci. 2021;22(9).

8. Pfaffl MW. A new mathematical model for relative quantification in real-time RT-PCR. Nucleic acids research. 2001;29(9):e45.

9. Yao J, Fang LC, Yang ZL, Huang H, Li Y, Deng J, et al. Mixed lineage leukaemia histone methylases 1 collaborate with ERalpha to regulate HOXA10 expression in AML. Biosci Rep. 2014;34(6):e00156.

10. Kuhn A, Loscher D, Marschalek R. The IRX1/HOXA connection: insights into a novel t(4;11)- specific cancer mechanism. Oncotarget. 2016;7(23):35341-52.

11. Cigdem S, Saito S, Nishikata D, Nagata K, Okuwaki M. SET-NUP214 and MLL cooperatively regulate the promoter activity of the HoxA10 gene. Genes to cells : devoted to molecular & cellular mechanisms. 2021;26(10):830-7.

12. Esposito MT, Zhao L, Fung TK, Rane JK, Wilson A, Martin N, et al. Synthetic lethal targeting of oncogenic transcription factors in acute leukemia by PARP inhibitors. Nat Med. 2015;21(12):1481-90.

13. Hijazi M, Smith R, Rajeeve V, Bessant C, Cutillas PR. Reconstructing kinase network topologies from phosphoproteomics data reveals cancer-associated rewiring. Nat Biotechnol. 2020;38(4):493-502.

14. Casado P, Rodriguez-Prados JC, Cosulich SC, Guichard S, Vanhaesebroeck B, Joel S, et al. Kinase-substrate enrichment analysis provides insights into the heterogeneity of signaling pathway activation in leukemia cells. Sci Signal. 2013;6(268):rs6.

15. Alcolea MP, Casado P, Rodriguez-Prados JC, Vanhaesebroeck B, Cutillas PR. Phosphoproteomic analysis of leukemia cells under basal and drug-treated conditions identifies markers of kinase pathway activation and mechanisms of resistance. Mol Cell Proteomics. 2012;11(8):453-66.

16. Montoya A, Beltran L, Casado P, Rodriguez-Prados JC, Cutillas PR. Characterization of a TiO(2) enrichment method for label-free quantitative phosphoproteomics. Methods. 2011;54(4):370-8.

17. Leeming MG, O'Callaghan S, Licata L, Iannuccelli M, Lo Surdo P, Micarelli E, et al. Phosphomatics: interactive interrogation of substrate-kinase networks in global phosphoproteomics datasets. Bioinformatics. 2021;37(11):1635-6.

18. Hornbeck PV, Zhang B, Murray B, Kornhauser JM, Latham V, Skrzypek E. PhosphoSitePlus, 2014: mutations, PTMs and recalibrations. Nucleic acids research. 2015;43(Database issue):D512-20.

19. Licata L, Lo Surdo P, Iannuccelli M, Palma A, Micarelli E, Perfetto L, et al. SIGNOR 2.0, the SIGnaling Network Open Resource 2.0: 2019 update. Nucleic acids research. 2020;48(D1):D504-D10.

20. Wiredja DD, Koyuturk M, Chance MR. The KSEA App: a web-based tool for kinase activity inference from quantitative phosphoproteomics. Bioinformatics. 2017;33(21):3489-91.

21. Huang da W, Sherman BT, Lempicki RA. Systematic and integrative analysis of large gene lists using DAVID bioinformatics resources. Nature protocols. 2009;4(1):44-57.

22. Sherman BT, Hao M, Qiu J, Jiao X, Baseler MW, Lane HC, et al. DAVID: a web server for functional enrichment analysis and functional annotation of gene lists (2021 update). Nucleic acids research. 2022;50(W1):W216-21.

23. Shannon P, Markiel A, Ozier O, Baliga NS, Wang JT, Ramage D, et al. Cytoscape: a software environment for integrated models of biomolecular interaction networks. Genome Res. 2003;13(11):2498-504.

24. Metsalu T, Vilo J. ClustVis: a web tool for visualizing clustering of multivariate data using Principal Component Analysis and heatmap. Nucleic acids research. 2015;43(W1):W566-70.

25. Gioutlakis A, Klapa MI, Moschonas NK. PICKLE 2.0: A human protein-protein interaction meta-database employing data integration via genetic information ontology. PloS one. 2017;12(10):e0186039.

26. Dobin A, Davis CA, Schlesinger F, Drenkow J, Zaleski C, Jha S, et al. STAR: ultrafast universal RNA-seq aligner. Bioinformatics. 2013;29(1):15-21.

27. Mortazavi A, Williams BA, McCue K, Schaeffer L, Wold B. Mapping and quantifying mammalian transcriptomes by RNA-Seq. Nat Methods. 2008;5(7):621-8.

28. Anders S, Huber W. Differential expression analysis for sequence count data. Genome Biol. 2010;11(10):R106.

29. Dillies MA, Rau A, Aubert J, Hennequet-Antier C, Jeanmougin M, Servant N, et al. A comprehensive evaluation of normalization methods for Illumina high-throughput RNA sequencing data analysis. Brief Bioinform. 2013;14(6):671-83.

30. Robinson MD, McCarthy DJ, Smyth GK. edgeR: a Bioconductor package for differential expression analysis of digital gene expression data. Bioinformatics. 2010;26(1):139-40.

31. Kanehisa M, Goto S. KEGG: kyoto encyclopedia of genes and genomes. Nucleic acids research. 2000;28(1):27-30.

32. Kim J-YY, Lee K-SS, Seol J-EE, Yu K, Chakravarti D, Seo S-BB. Inhibition of p53 acetylation by INHAT subunit SET/TAF-Iβ represses p53 activity. Nucleic acids research. 2012;40(1):75-87.
