# Supplementary material for: SET-PP2A complex as a new therapeutic target in KMT2A (MLL) rearranged AML
Source: Oncogene. 2023 Oct 27;42(50):3670–83. doi: 10.1038/s41388-023-02840-1 (PMC10709139; doi:10.1038/s41388-023-02840-1)

Supplementary Figure 1

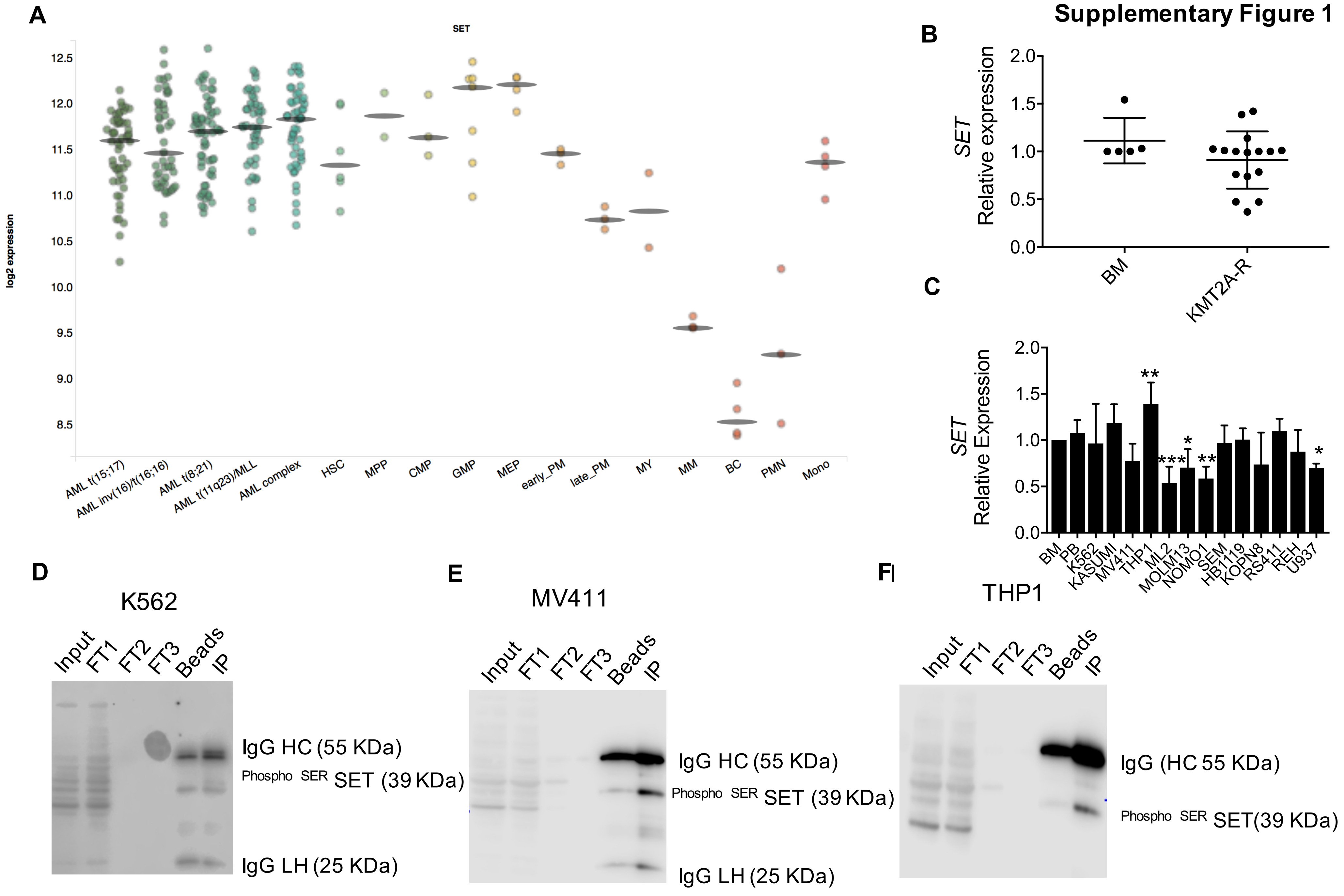

Supplement: Supplementary file 6 — Supplementary Figure 1 [file 41388_2023_2840_MOESM6_ESM.pdf]
