# Supplementary figures and images for: SET-PP2A complex as a new therapeutic target in KMT2A (MLL) rearranged AML
Source: Oncogene. 2023 Oct 27;42(50):3670–83. doi: 10.1038/s41388-023-02840-1 (PMC10709139; doi:10.1038/s41388-023-02840-1)

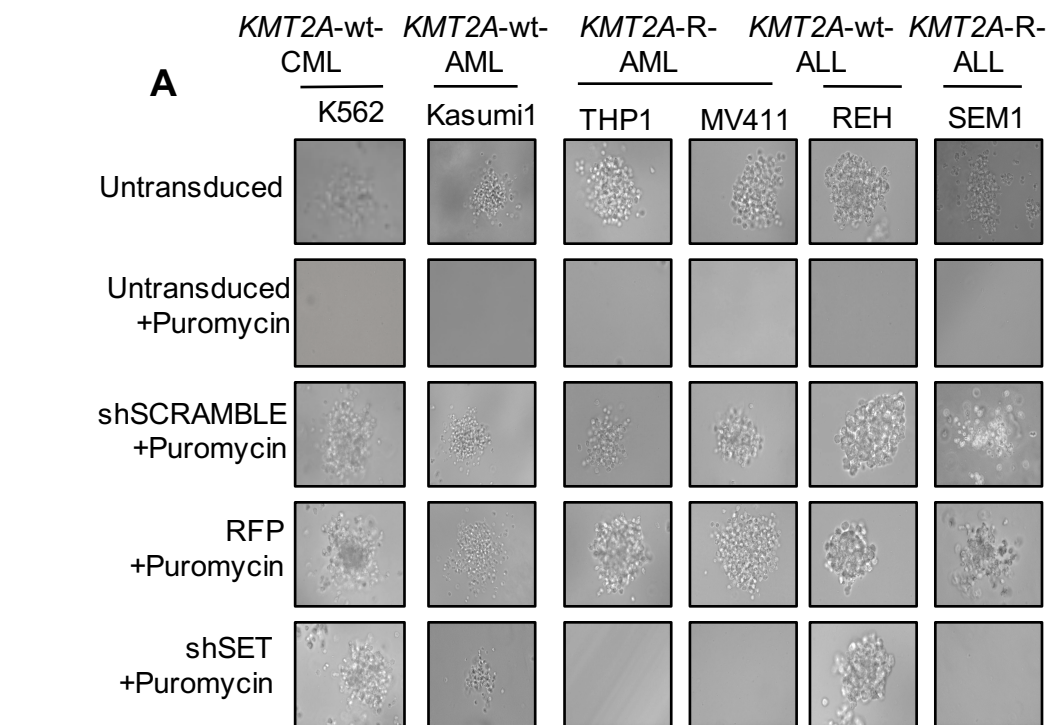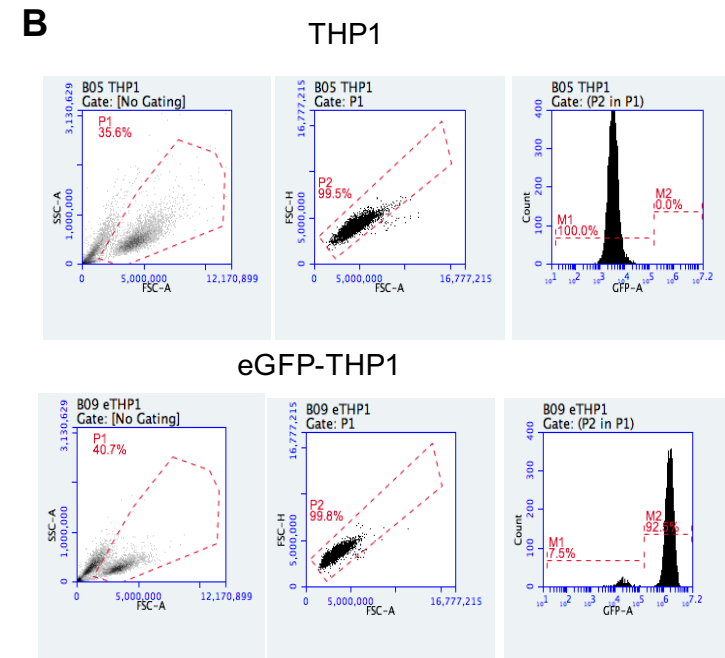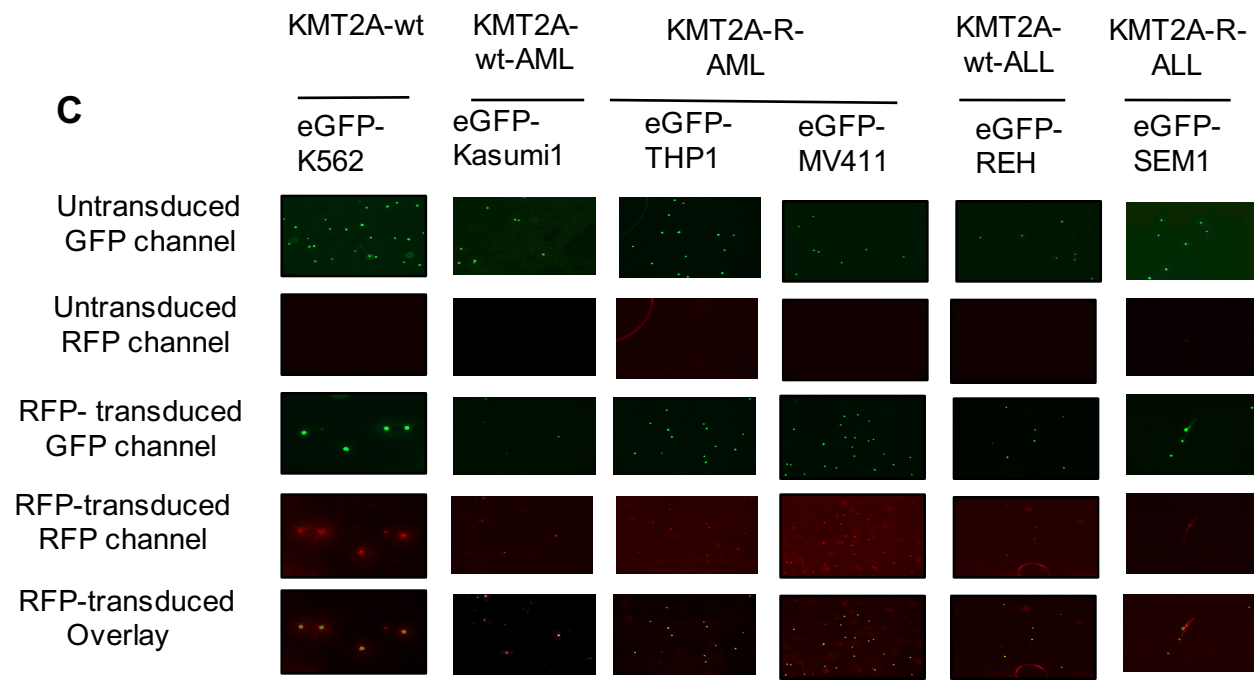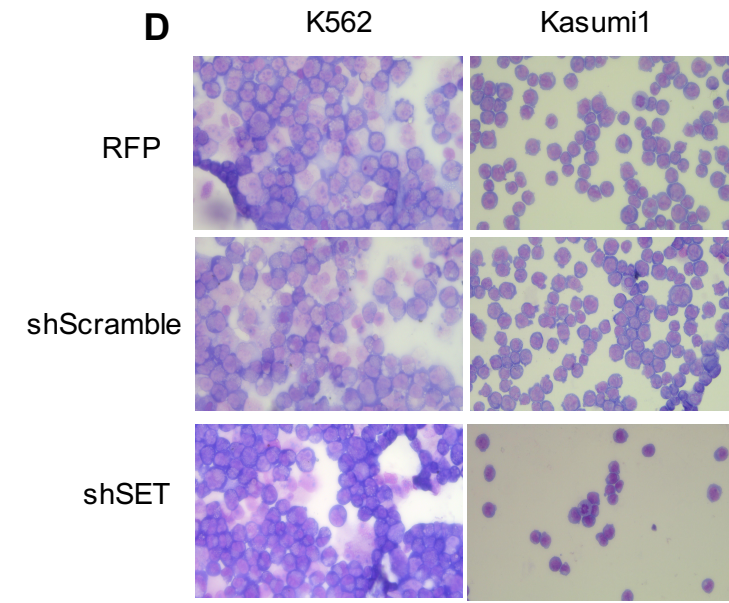

Supplement: Supplementary file 7 — Supplementary Figure 2 [file 41388_2023_2840_MOESM7_ESM.pdf]

# Supplementary Figure 3

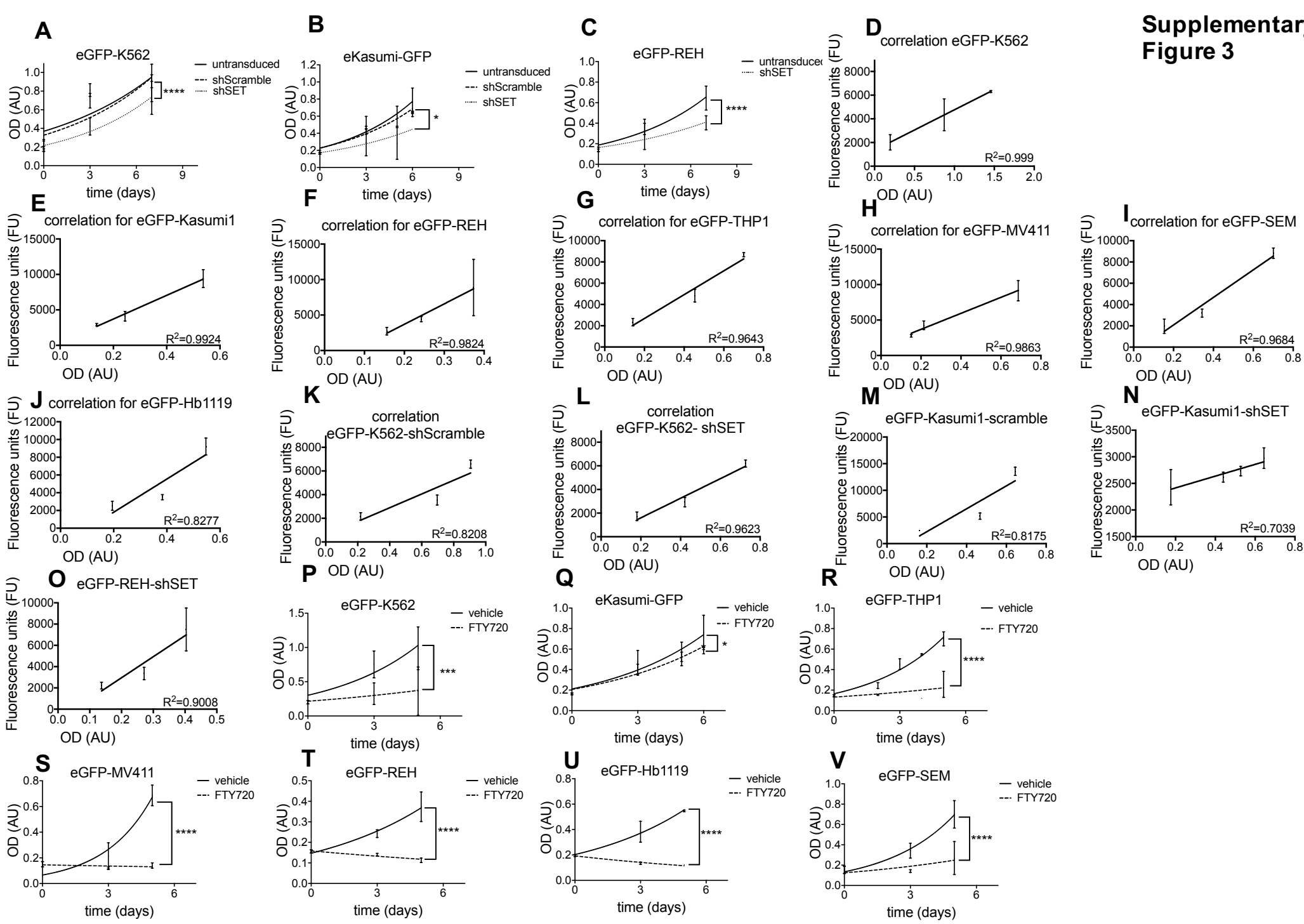

Supplement: Supplementary file 8 — Supplementary Figure 3 [file 41388_2023_2840_MOESM8_ESM.pdf]

A

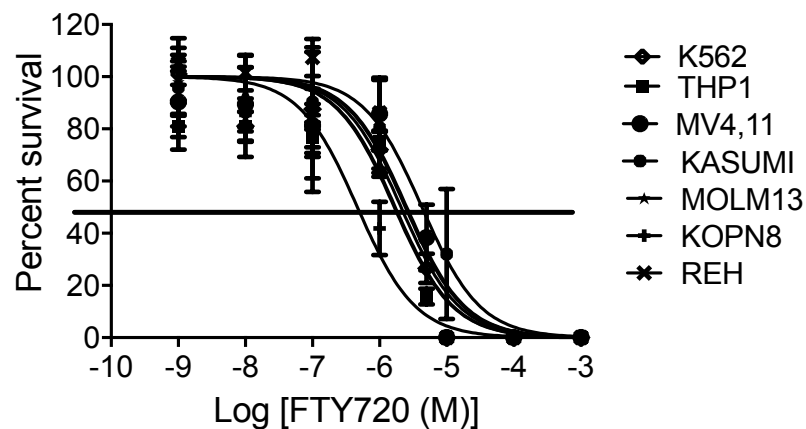

B

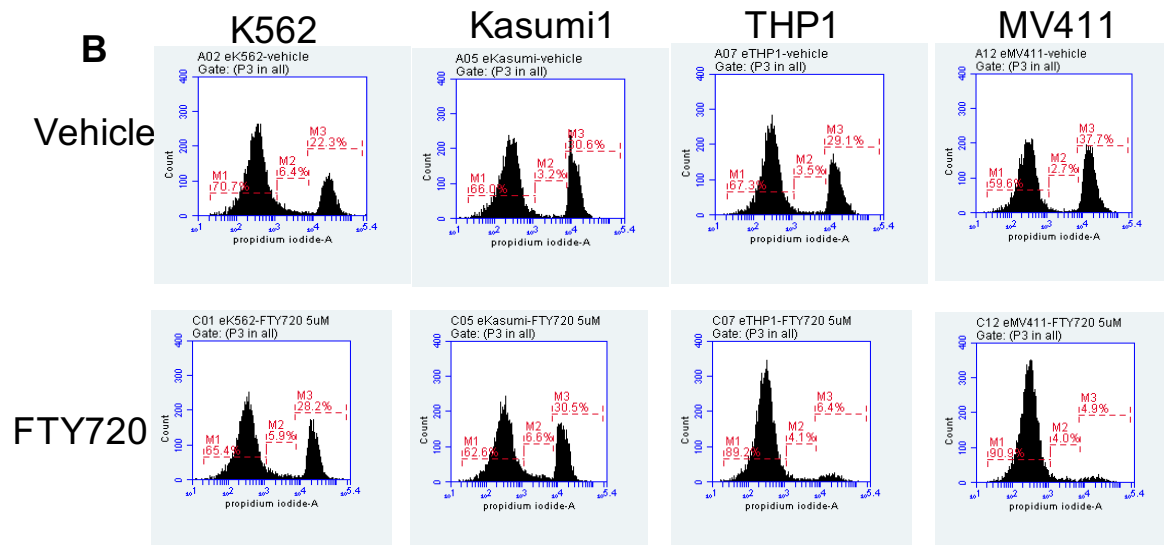

C

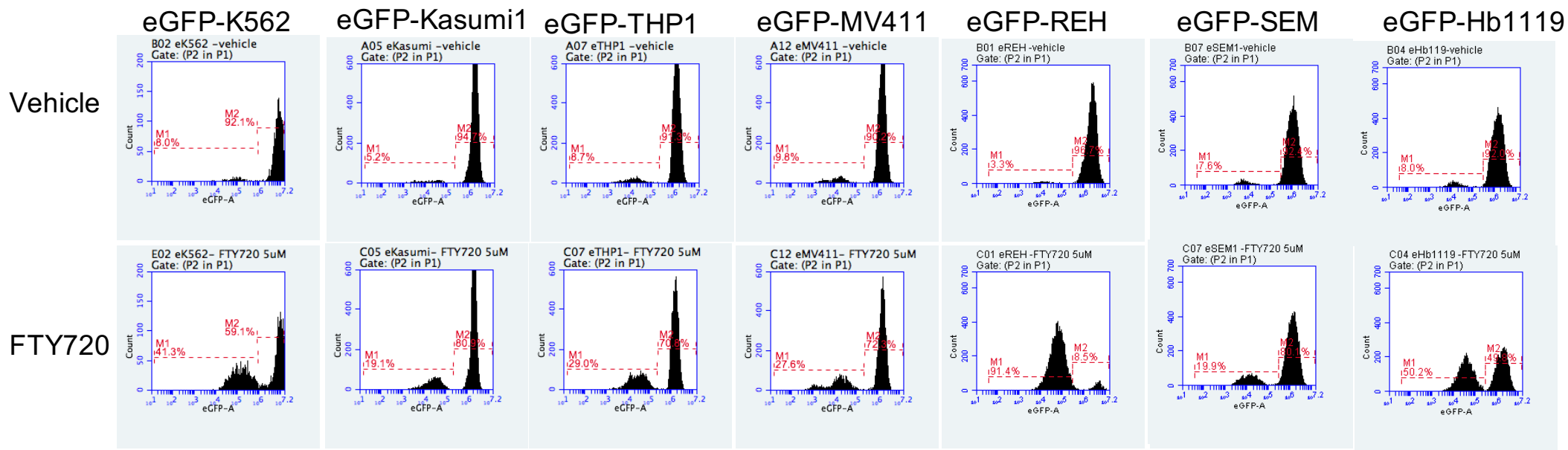

Supplement: Supplementary file 9 — Supplementary Figure 4 [file 41388_2023_2840_MOESM9_ESM.pdf]

**A**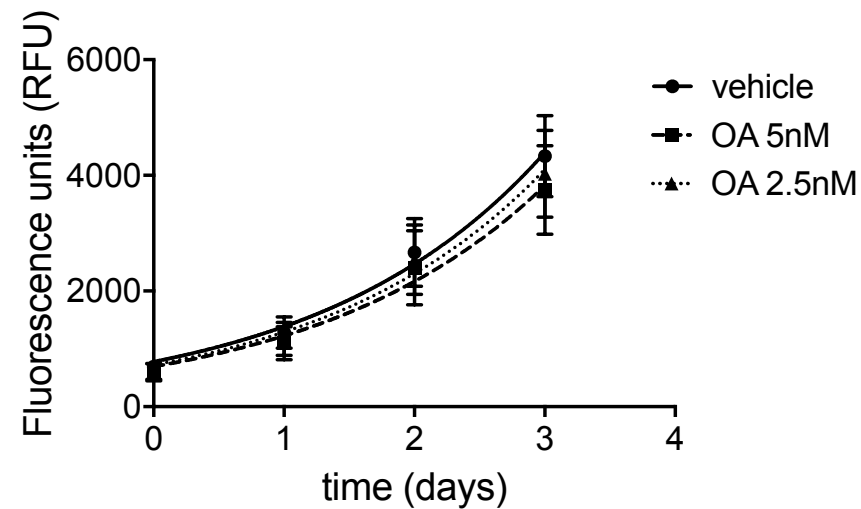**B**

K562

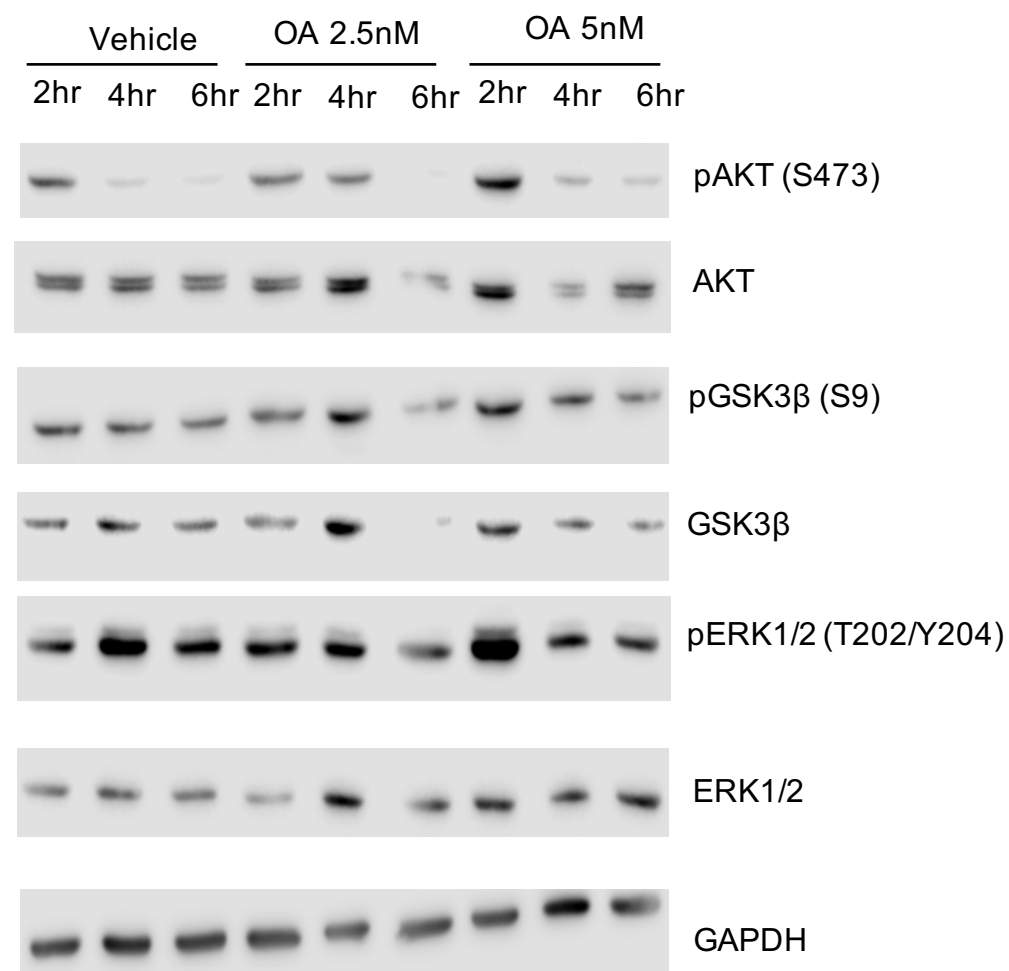**C**

K562

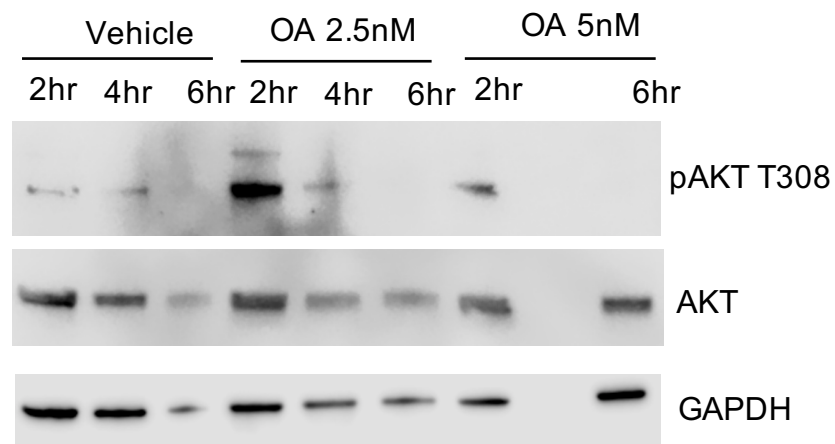

Supplement: Supplementary file 10 — Supplementary figure 5 [file 41388_2023_2840_MOESM10_ESM.pdf]

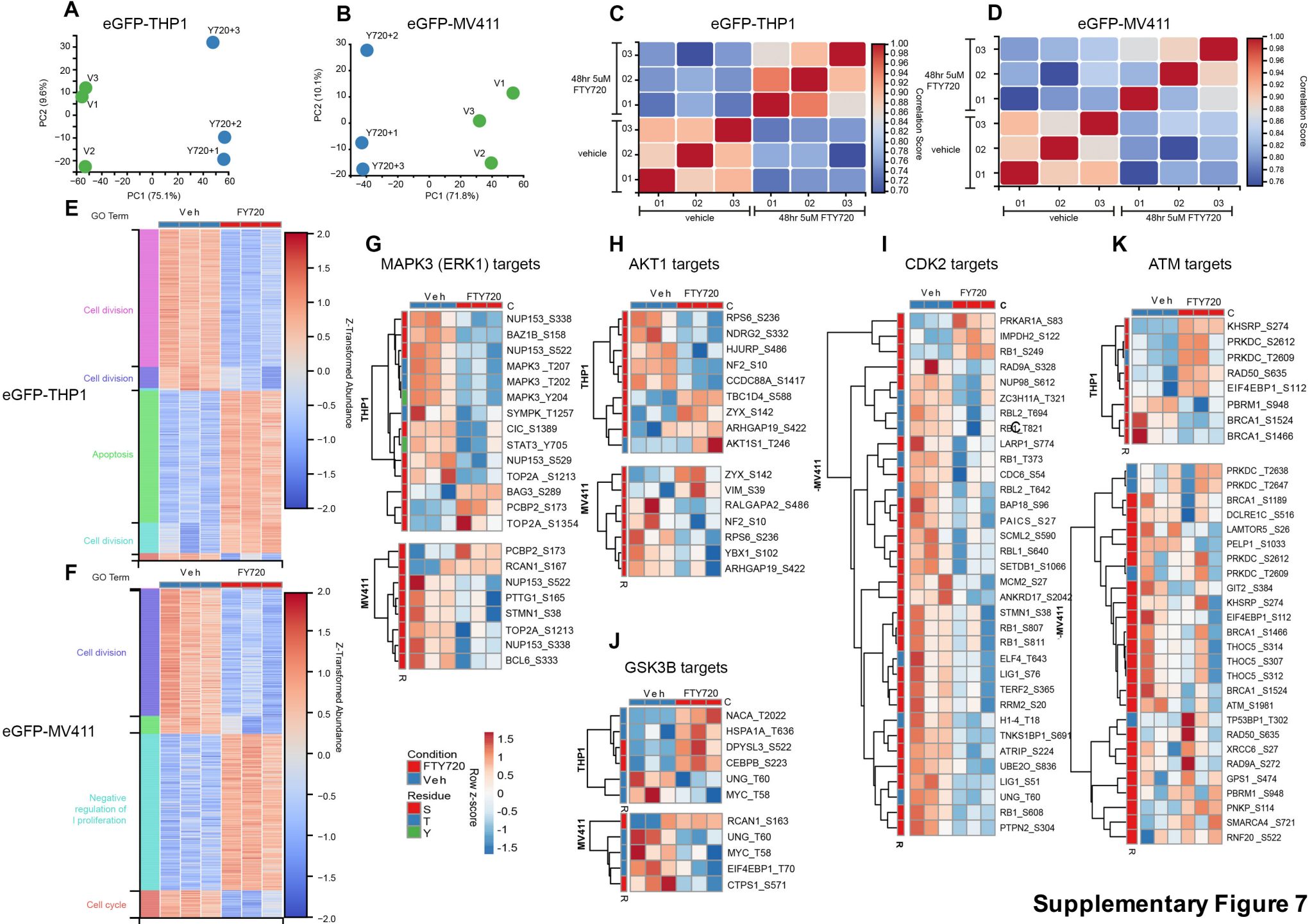

Supplementary Figure 7

Supplement: Supplementary file 12 — Supplementary Figure 7 [file 41388_2023_2840_MOESM12_ESM.pdf]

Supplementary Figure 8

**A**

## FTY720vsDMSO.down(GO)

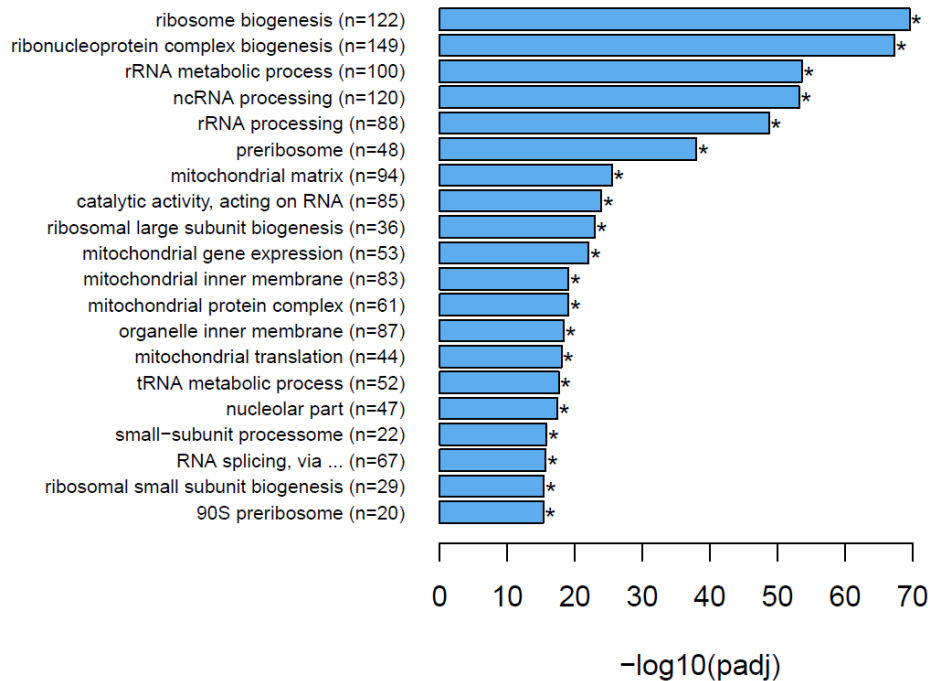**B**

## FTY720vsDMSO.up(GO)

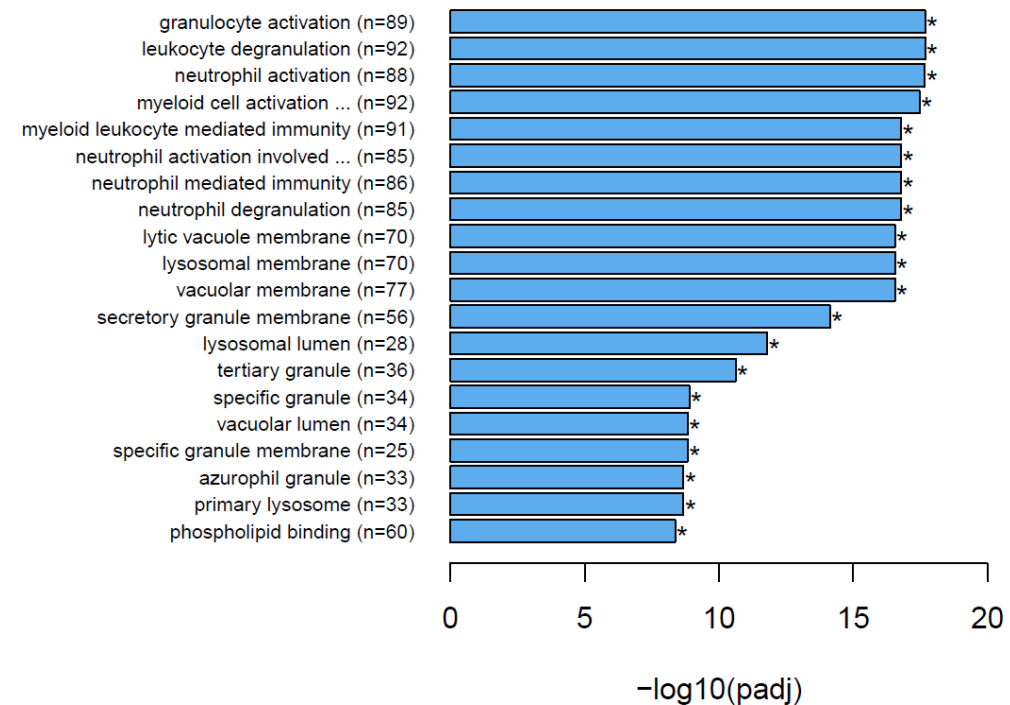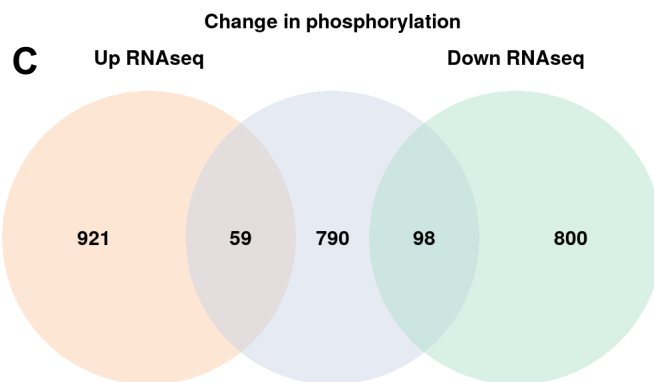**D**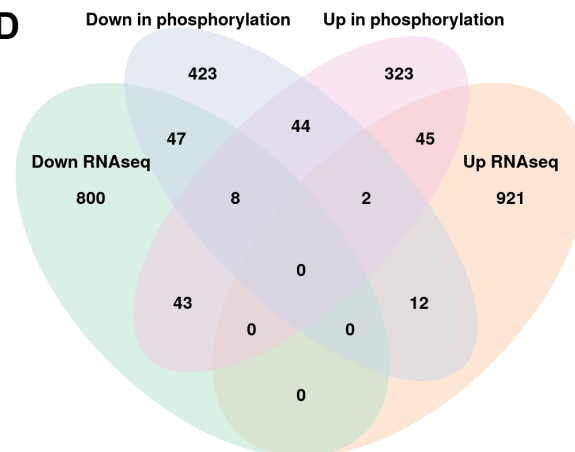

Supplement: Supplementary file 13 — Supplementary Figure 8 [file 41388_2023_2840_MOESM13_ESM.pdf]

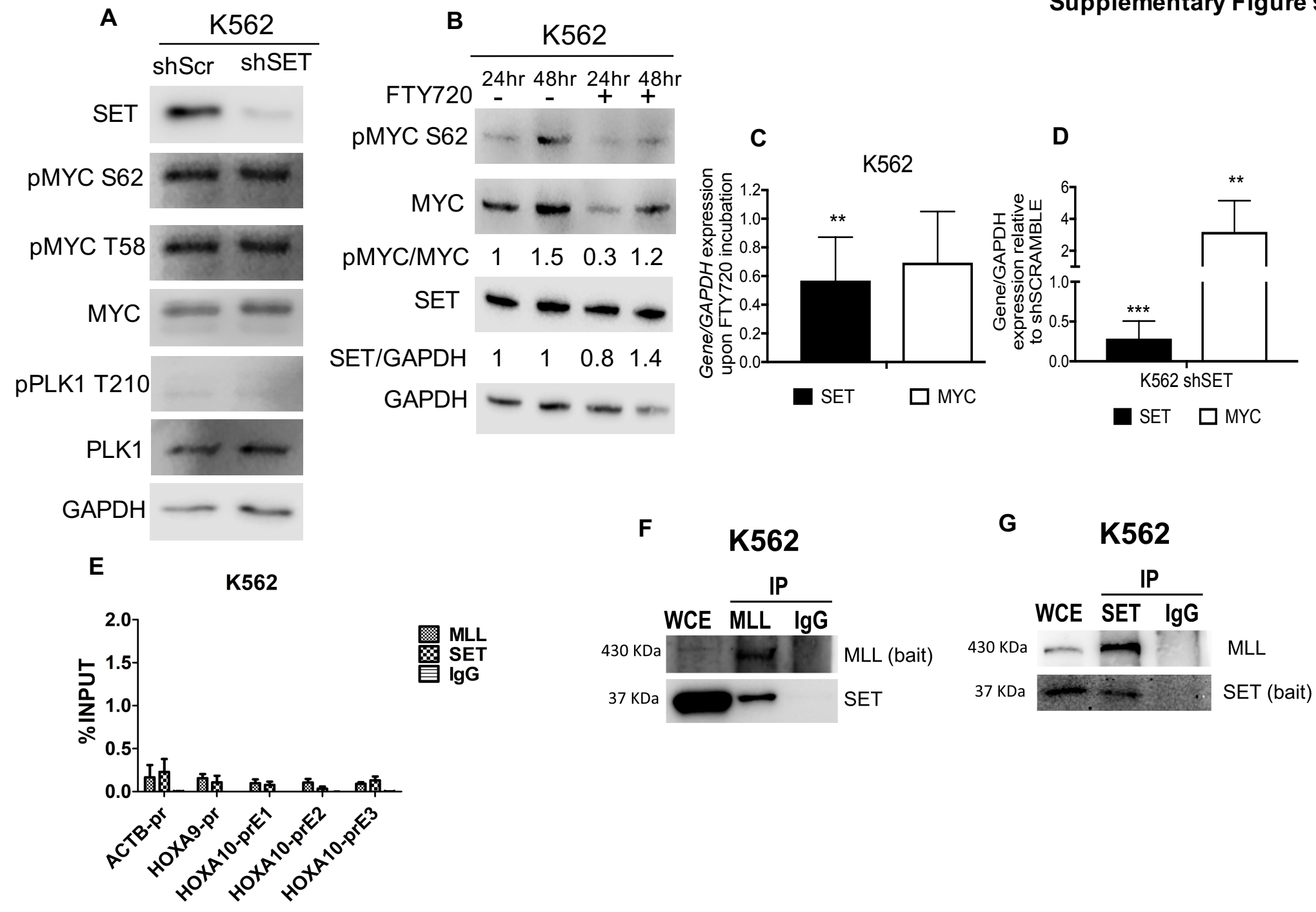

Supplement: Supplementary file 14 — Supplementary Figure 9 [file 41388_2023_2840_MOESM14_ESM.pdf]

Supplementary Figure 10

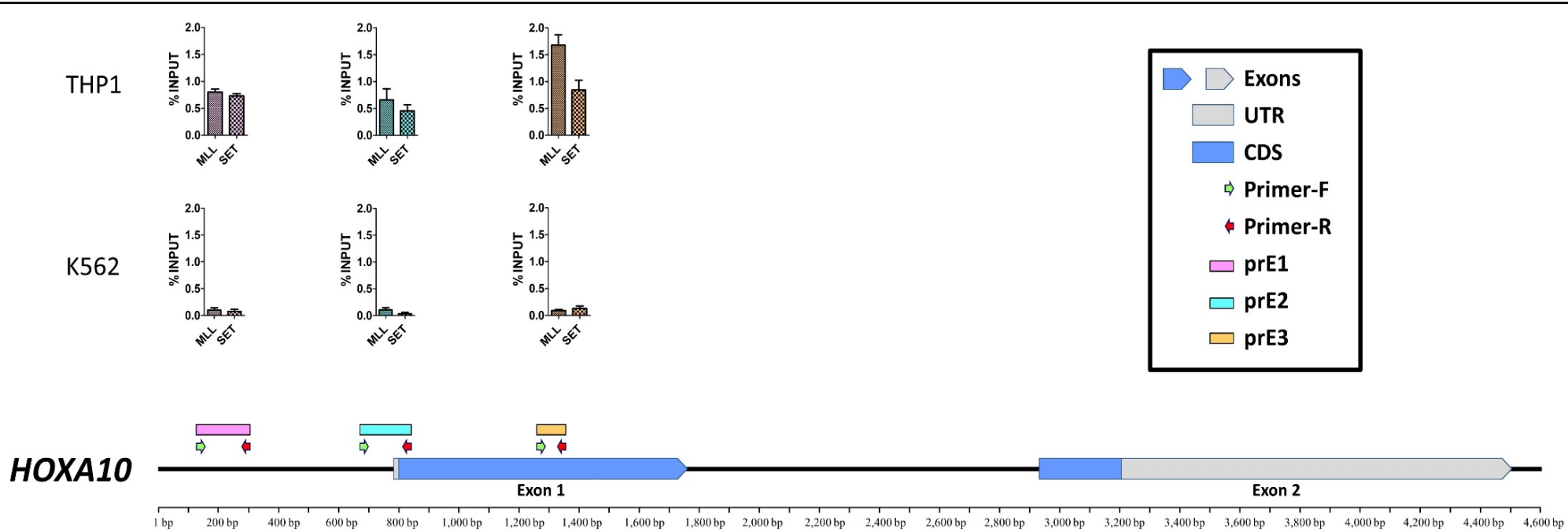

Supplement: Supplementary file 15 — Supplementary Figure 10 [file 41388_2023_2840_MOESM15_ESM.pdf]
